# Human polyomavirus BKV infection of endothelial cells results in interferon pathway induction and persistence

**DOI:** 10.1371/journal.ppat.1007505

**Published:** 2019-01-08

**Authors:** Ping An, Maria Teresa Sáenz Robles, Alexis M. Duray, Paul G. Cantalupo, James M. Pipas

**Affiliations:** Department of Biological Sciences, University of Pittsburgh, Pittsburgh, Pennsylvania, United States of America; University of Michigan, UNITED STATES

## Abstract

Polyomavirus BKV is highly prevalent among humans. The virus establishes an asymptomatic persistent infection in the urinary system in healthy people, but uncontrolled productive infection of the virus in immunocompromised patients can lead to serious diseases. In spite of its high prevalence, our knowledge regarding key aspects of BKV polyomavirus infection remains incomplete. To determine tissue and cell type tropism of the virus, primary human epithelial cells, endothelial cells and fibroblasts isolated from the respiratory and urinary systems were tested. Results from this study demonstrated that all 9 different types of human cells were infectable by BKV polyomavirus but showed differential cellular responses. In microvascular endothelial cells from the lung and the bladder, BKV persistent infection led to prolonged viral protein expression, low yield of infectious progeny and delayed cell death, in contrast with infection in renal proximal tubular epithelial cells, a widely used cell culture model for studying productive infection of this virus. Transcriptomic profiling revealed the activation of interferon signaling and induction of multiple interferon stimulated genes in infected microvascular endothelial cells. Further investigation demonstrated production of IFNβ and secretion of chemokine CXCL10 by infected endothelial cells. Activation of IRF3 and STAT1 in infected endothelial cells was also confirmed. In contrast, renal proximal tubular epithelial cells failed to mount an interferon response and underwent progressive cell death. These results demonstrated that microvascular endothelial cells are able to activate interferon signaling in response to polyomavirus BKV infection. This raises the possibility that endothelial cells might provide initial immune defense against BKV infection. Our results shed light on the persistence of and immunity against infection by BKV polyomavirus.

## Author summary

Infection by polyomavirus BKV is common and mostly harmless in healthy populations but can cause severe damages to kidney and bladder in transplant recipients. The infection by BKV usually occurs in early childhood and persists chronically in the urinary system throughout life. Our data show that this virus has the ability to infect multiple types of human cells along the respiratory and urinary tracts. Furthermore, the infection elicits an immune response in endothelial cells, the type of cells that line the inner surface of the blood vessels. These results provide insights into the distinct cellular responses displayed by different cell types that BKV encounters during infection and spread of the virus within the body, and on innate immune responses against the infection.

## Introduction

Infection of BK polyomavirus (BKV) in humans is widespread with seroprevalence ranging from 60 to over 90% in populations world-wide [1–3]. Seroconversion of BKV occurs in early childhood and the lifelong infection persists asymptomatically in most individuals [2, 3]. The site and entry route of initial BKV infection and dissemination route of the virus, as well as the mode of transmission remain to be determined. Diseases associated with BKV only affect immunocompromised populations, especially transplant recipients and AIDS patients, thus host immunity against BKV is critical in limiting pathological consequences due to productive infection by the virus. Immune defense against BKV infection involves both humoral and cellular immunity [4–7], however, our knowledge regarding BKV specific immunity at the molecular level is limited.

Two observations suggest that persistence of BKV is maintained in the urinary system. First, BKV is occasionally detected in urine from healthy individuals; second, productive BKV infection is associated with nephropathy and hemorrhagic cystitis in transplant patients. However, several studies indicate a wide spectrum of BKV tissue and cell type tropism. For instance, most of the 60 NCI-60 tumor cell lines are transducible with BKV pseudovirions encapsidating either a GFP or luciferase reporter [8]. In addition, BKV has been associated with HIV-associated salivary gland disease [9–11], and replication of BKV has been demonstrated in human salivary gland cells [10]. BKV has also been shown to infect human peripheral blood leukocytes and pancreatic cells [12, 13], and animal experiments have demonstrated the uptake of blood born BKV virus-like particles by endothelial cells [14]. Finally, HUVEC (human umbilical cord vein endothelial cells) support the growth of archetype BKV [15] and BKV infection was detected in vascular endothelial cells of a renal transplant recipient patient exhibiting vasculopathy [16]. Thus, while it is clear BKV sometimes replicates in the urinary system, especially under pathologic conditions, the site of persistence is unknown.

Primary human RPTE (renal proximal tubule epithelial cells) provide a valuable and widely used *in vitro* model for understanding BKV infection [17, 18]. RPTE support productive infection of the virus and many aspects of BKV infection have been studied using this system [17, 19–23]. Under culture conditions, BKV undergoes a complete productive infection of RPTE, leading to the release of many progeny virions and loss of cell monolayer viability. This is not surprising given that RPTE do not elicit an efficient innate immune response against BKV infection [20, 23, 24]. Two separate transcriptomic profiling studies, using microarray and RNA-seq respectively, did not detect changes in gene expression indicative of activated IFN signaling [20, 23] and cytokine profiling further confirmed a lack of pro-inflammatory responses following BKV infection [24]. In contrast, infection of RPTE with influenza A virus, herpes simplex virus 1, cytomegalovirus and the closely related human polyomavirus JCV do produce robust antiviral responses indicating that these cells are fully capable of detecting and responding to viral pathogens [23, 24]. However, BKV is able to trigger an innate immune response, as indicated by the elevated levels of CXCL10 and ZBP1, released by leukocytes in response to BKV infection [24].

Taken together, these results suggest that RPTE do not mount an effective antiviral response to counter BKV infection, thus renal proximal tubular cells may not be responsible for triggering the acquired immunity that limits BKV productive infection *in vivo*. Furthermore, the robust productive infection exhibited by RPTE make it unlikely that persistence is maintained in these cells. In this study, we have found that BKV infection of microvascular endothelial cells (VEC) induces a significant innate immune response and that BKV infection persists in these cells for several weeks. These results expand our knowledge of the differential susceptibility and response of specific cells to BKV infection, and provide important implications regarding BKV-induced specific immunity and persistence.

## Results

### BKV infects multiple human cell-types

To survey BKV infectivity in human tissues, we obtained 9 types of primary human cells isolated from the lung and urinary systems through commercial sources. These included epithelial cells from lung, proximal tubule and medulla of kidney, bladder and urethra, fibroblasts from lung and bladder, and microvascular endothelial cells from lung and bladder. Cells were inoculated with BKV stock at a MOI (multiplicity of infection) of 1 FFU (fluorescence forming units)/cell and expression of BKV T antigens (TAg) and VP1 were examined by IF (immunofluorescence staining) using specific antibodies.

**Fig 1:**
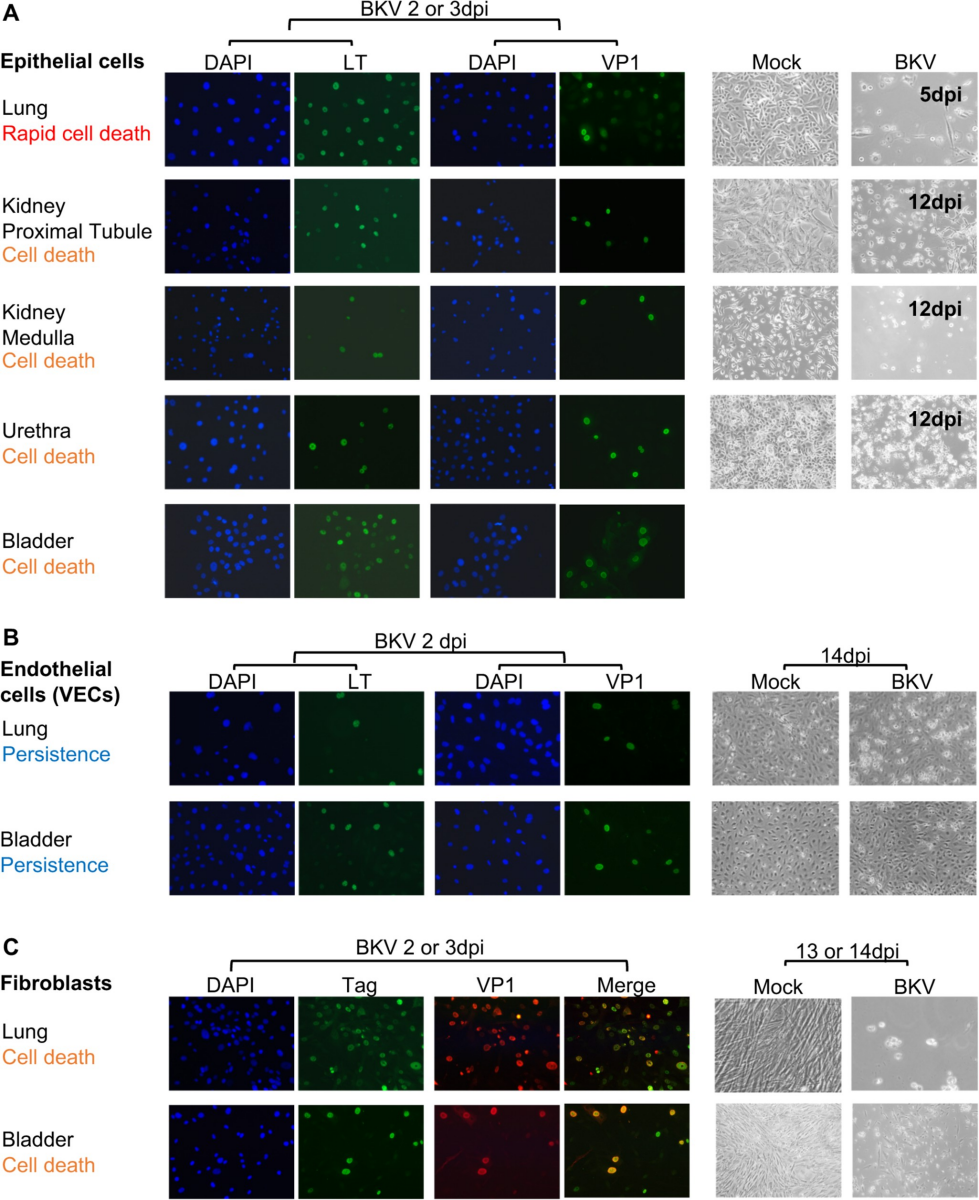
Infection of BKV in 9 types of human primary cells. **A.** BKV infection of epithelial cells from lung, kidney, bladder and urethra. **B.** BKV infection of endothelial cells from lung and bladder. For A and B, images for positive staining of LT (Pab 416) and VP1 in BKV inoculated cells at 2 or 3dpi are shown as indicated. The matching DAPI staining for each field are included. **C.** BKV infection in fibroblasts from lung and bladder. Co-staining images of TAg (GaT) and VP1 are shown separately and then as merged images as indicated. Pictures of live cells are shown next to the IF images and times (dpi) at which pictures were taken are indicated. The organ source for each cell type and outcome of infection is listed on the left of each panel.

All cell types showed expression of both TAg and VP1 at 2 or 3dpi (days post inoculation) (Fig 1). Co-staining showed overlapping TAg and VP1 signals in infected fibroblasts from both lung and bladder, indicating that most cells expressing TAg also produced VP1, thus suggesting productive infection. We also observed the cytopathic effect (CPE) induced by BKV in each cell type. Six cell types, including epithelial cells from kidney, bladder and urethra, and the fibroblasts from the lung and bladder, underwent near complete cell death by about 14dpi (Fig 1A and 1C, live cell images). Epithelial cells from the lung developed the most rapid CPE, where most cells died and detached before 5dpi (Fig 1A). In contrast, CPE in the inoculated vascular endothelial cells was less obvious and many cells still survived at 14dpi (Fig 1B). These results were confirmed using RPTE, lung epithelial cells and lung endothelial cells from additional donors. Specific donor information and number of donors tested are listed in S1 Table. We conclude that all 9 cell-types tested can be infected by BKV, but the effects of infection on cell viability are less pronounced in endothelial cells.

### BKV infection is limited in vascular endothelial cells

To further understand the limited CPE observed in microvascular endothelial cells, referred to as VEC in the rest of the manuscript, we directly compared the responses to BKV in VEC to those in RPTE, a well-established cell culture model for studying BKV productive infection. RPTE, LVEC (lung VEC) and BVEC (bladder VEC) were inoculated with BKV and cell number, viral transcription and protein expression, as well as release of progeny virions were monitored at various times post-inoculation. Two RPTE, three LVEC and one BVEC, each from an independent donor, were used in these experiments.

**Fig 2:**
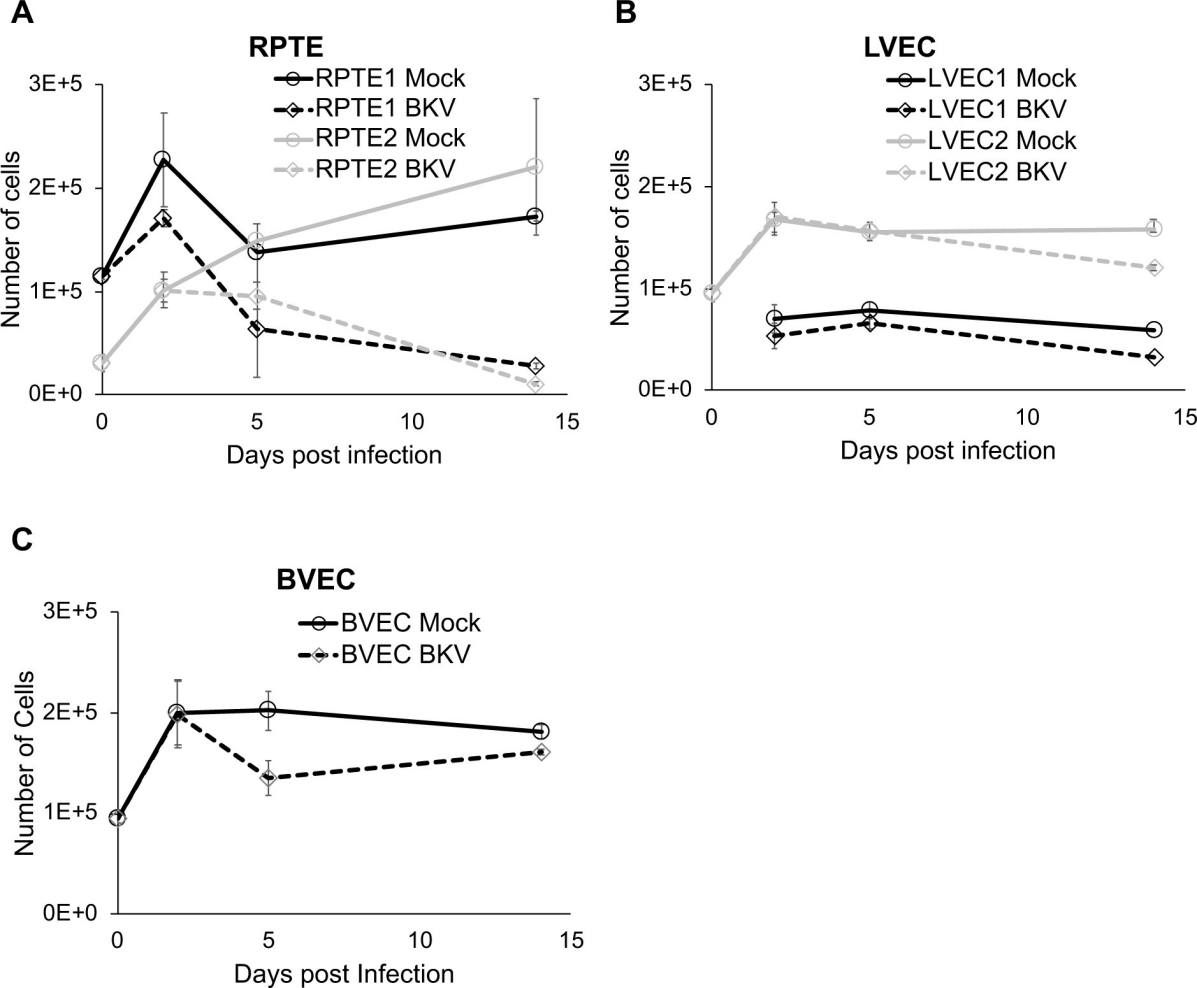
Cell death in BKV infected RPTE and VEC. Changes in cell numbers of RPTE (**A**), LVEC (**B**) and BVEC (**C**) at 0, 2, 5 and 14dpi. Results for RPTE and LVEC from two donors each (1 in black and 2 in gray) are shown. Day 0, the day of inoculation. Mock (solid lines); BKV inoculated (dashed lines). Error bars represent standard deviation of three trials.

**Fig 3:**
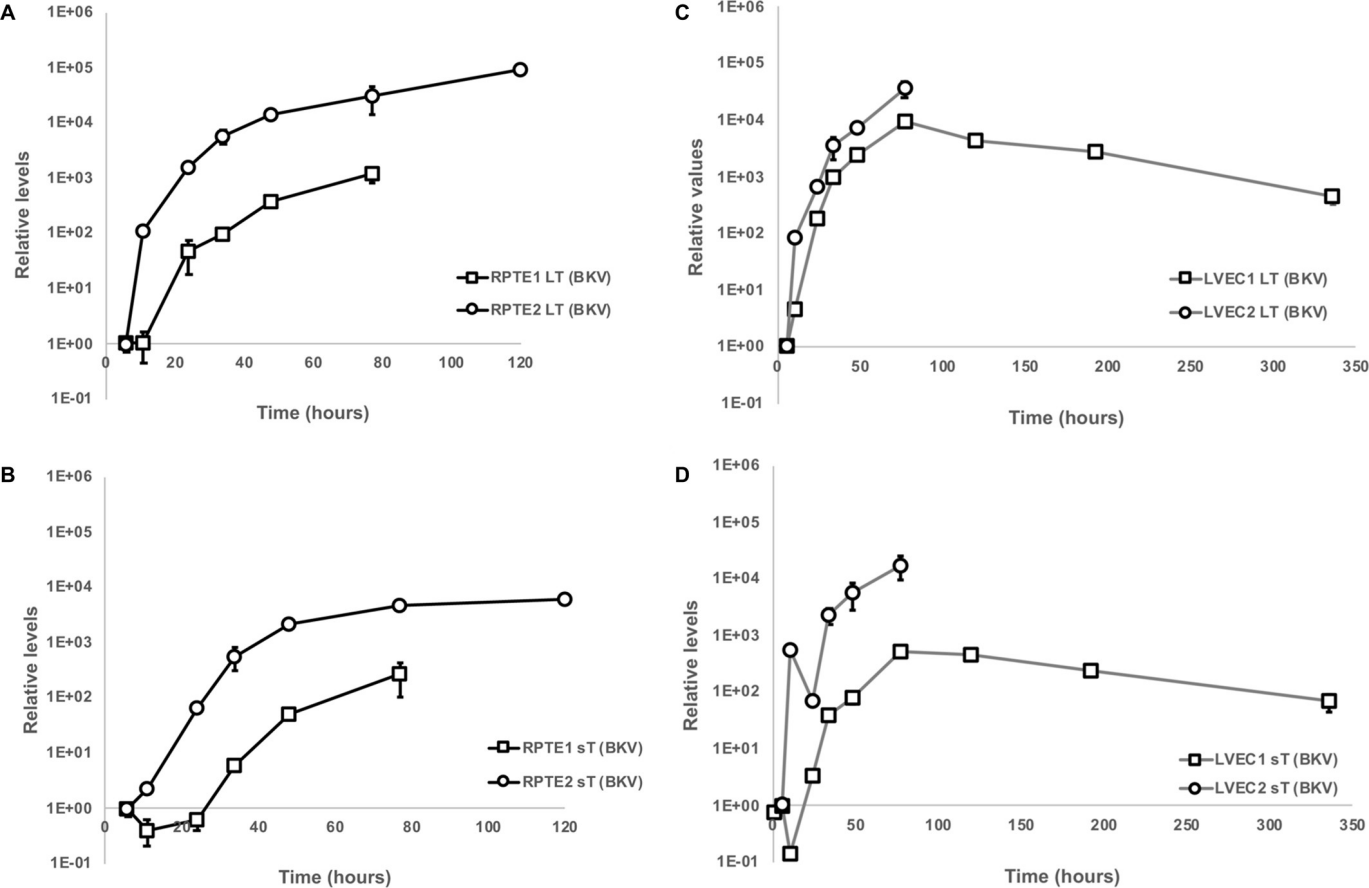
Expression of LT and sT in RPTE and LVEC. Levels of LT and sT mRNAs were measured with specific primers by RT-qPCR analysis. Two donors for each RPTE and LVEC were evaluated. **A** and **B**, expression of LT and sT, respectively, in mock and BKV inoculated RPTE cells; **C** and **D**, expression of LT and sT, respectively, in mock and BKV inoculated LVEC cells. Error bars represent standard deviation. The levels of LT and sT in mock at all tested timepoints were below background levels of BKV inoculated samples at 0h.

**Fig 4:**
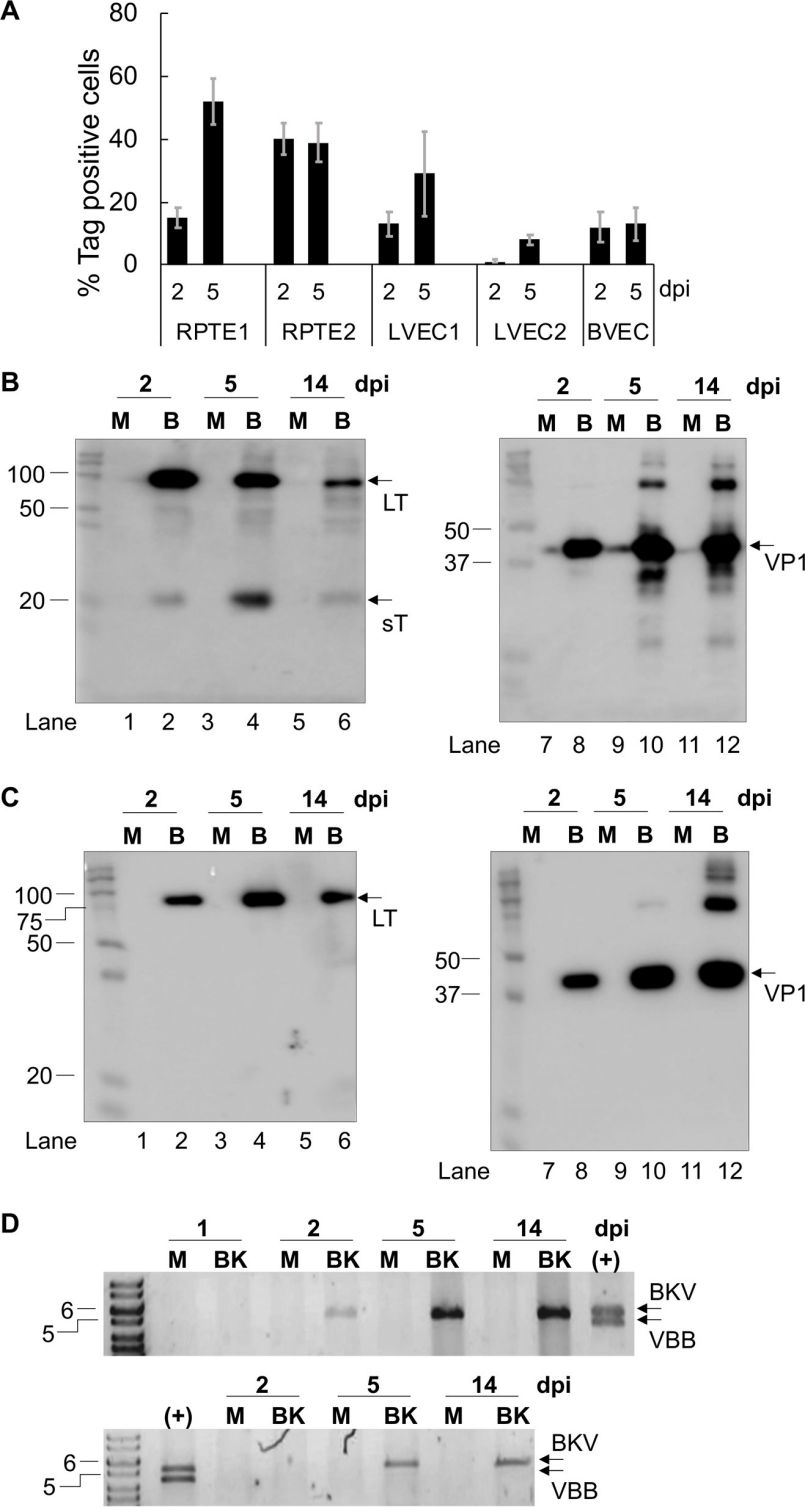
Viral protein expression and DNA replication in RPTE and LVEC. **A.** Quantification of % LT positive cells in BKV inoculated RPTE and VEC using IF at 2 and 5dpi. Error bars represent standard deviation of three measurements. **B.** Western blots showing TAg (left panel) and VP1 (right panel) expression in mock and BKV inoculated RPTE2 at 2, 5 and 14dpi. Molecular weights (kDa) of the standards are indicated on the left side of each panel. Arrows indicate LT, sT and VP1 proteins. M, mock (Lanes 1, 3, 5, 7, 9 and 11); B, BKV (Lanes 2, 4, 6, 8, 10, 12). **C.** Western blots showing LT (left panel) and VP1 (right panel) expression in mock and BKV inoculated LVEC2 at 2, 5 and 14dpi. **D.** Agarose gel image of BKV genomic DNA isolated from mock (M) and BKV (BK) inoculated RPTE1 (upper panel) and LVEC2 (lower panel). The 5kb and 6kb bands of the molecular weight markers are indicated on the left side of the panel. (+), positive control using digested pBKV plasmid, which produced two bands. Upper band (BKV), linearized BKV genomic DNA; lower band (VBB), plasmid vector backbone.

In RPTEs from both donors, cell number differences between the mock and BKV inoculated conditions grew larger as the infection progressed from 2 to 14 dpi (Fig 2A). By 14dpi, the cell numbers of mock-inoculated RPTEs increased a modest amount, while cell number of inoculated cells dropped dramatically. In contrast, the reduction in cell numbers following BKV infection was moderate in both LVEC and BVEC (Fig 2B and 2C). We next investigated viral gene expression at the RNA and protein levels. Transcripts for large T antigen (LT) or small T antigen (sT), were monitored by RT-qPCR (Fig 3). LVEC expressed both LT and sT RNAs at levels comparable to RPTE (Fig 3C and 3D). The expression of viral T antigens in LVEC1 was followed for 2 weeks, during which LT and sT expression peaked around 3dpi and then decreased gradually over the next 10 days. At 14dpi, LT and sT expressions decreased about 13 or 8-fold from their respective peak levels. Thus, the timing and the levels of viral early gene expression in both RPTE and LVEC were similar. Examination of LT by immunofluorescence showed that at 2dpi the percentage of LT positive cells was similar in RPTE1, RPTE2, LVEC1, and BVEC but lower in LVEC2 (Fig 4A). However, at 5dpi there were fewer LT-positive cells in all three VECs compared to both RPTEs. Quantification of positive cells was not attempted at 14dpi due to high background in the staining resulting from cell debris. The expression of LT and VP1 in mock and BKV inoculated RPTE2 and LVEC2 was also verified by Western blot, using specific anti TAg and anti-VP1 antibodies (Fig 4B and 4C). Both proteins were readily detectable in inoculated cells at all tested time points.

**Fig 5:**
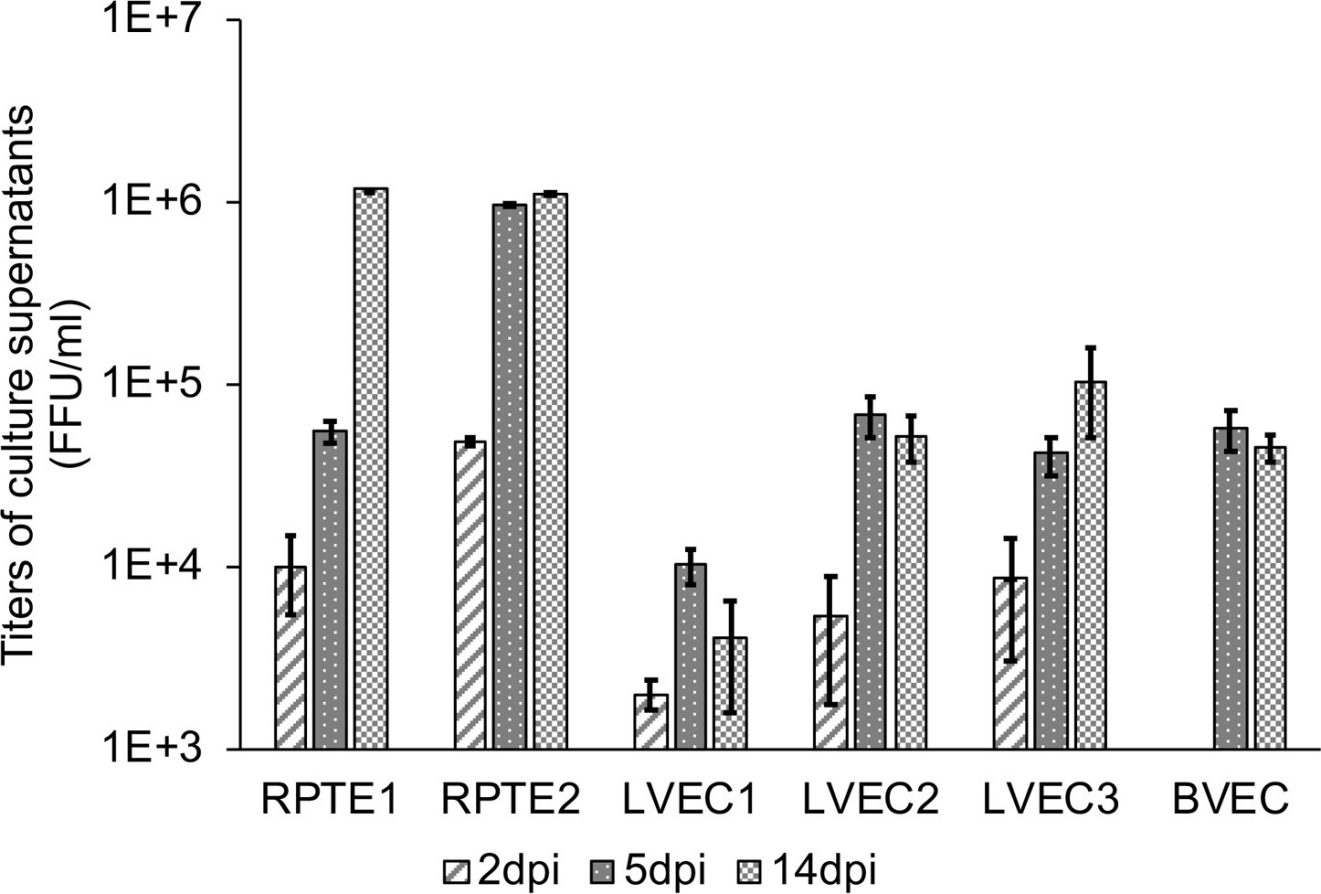
BKV infection in RPTE and VEC led to production of infectious progeny viruses. Titration of cell culture supernatants collected at 2, 5 and 14dpi. Note the minimum value for the Y-axis starts at 1E+3. Error bars represent standard deviation of three measurements.

To evaluate viral DNA replication, DNA was isolated and characterized as described in materials and methods. BKV genomic DNA was first detected at 2dpi in inoculated RPTE, and the amount of DNA increased by 5dpi and 14dpi (Fig 4D, upper panel). In contrast, BKV DNA was not detectable until 5dpi in LVEC2 (Fig 4D, lower panel). Furthermore, we observed that the amount of BKV DNA in LVEC2 was lower than that in RPTE1. Additional experiments showed similar differences between LVEC1 and RPTE2 (S1 Fig). Next, we measured the production of infectious progeny virus for all RPTEs and VECs by titration of the culture supernatants collected at 2, 5 and 14dpi. The titers of VEC supernatants were consistently lower than those of RPTE supernatants at all time points (Fig 5). At 14dpi, when the RPTE-inoculated monolayer was destroyed, viral titers from infected VECs were roughly 10 to 270-fold less than those from RPTEs. These results indicate that BKV infection is limited in VEC relative to RPTE.

### BKV infection persists in vascular endothelial cells

**Fig 6:**
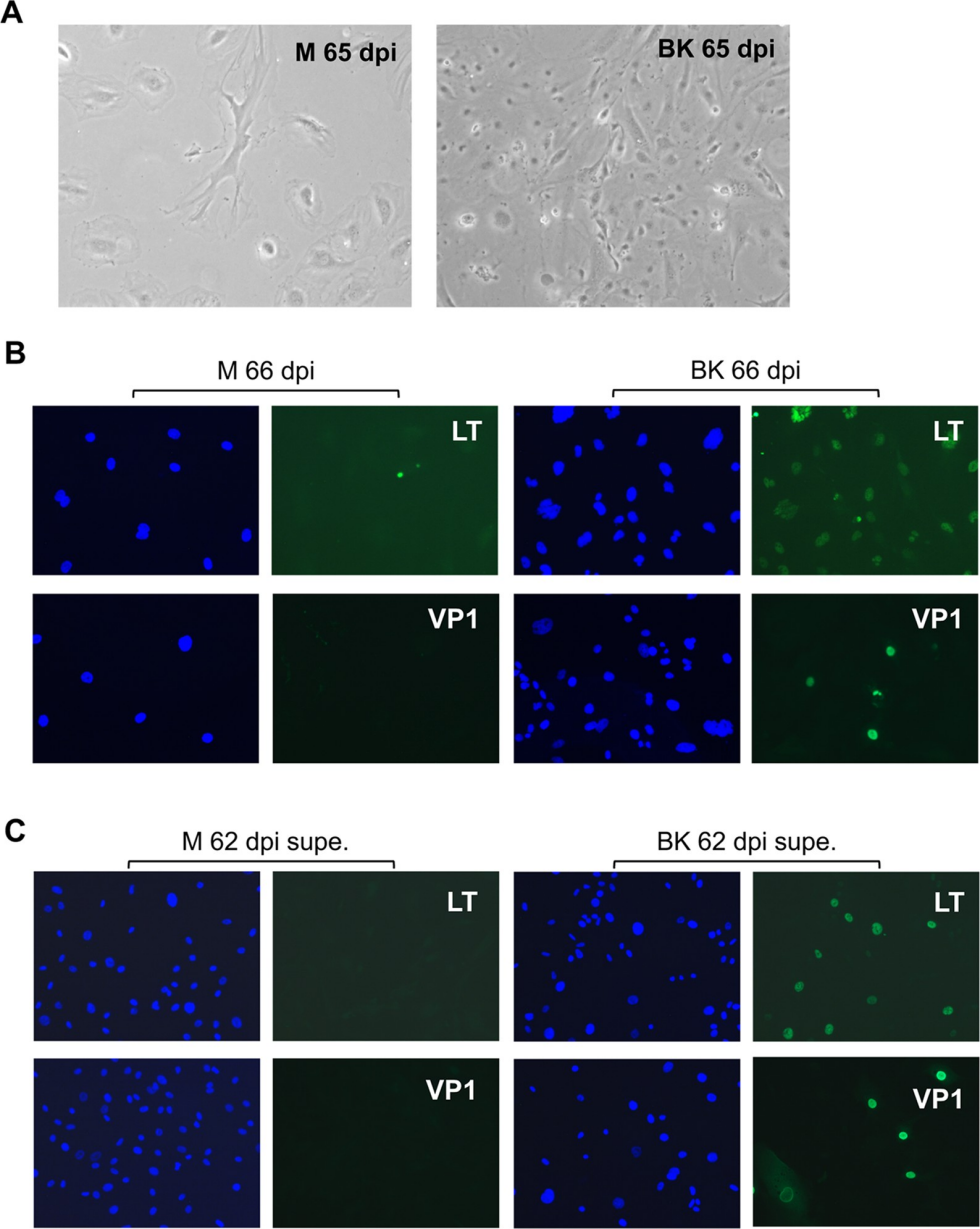
Persistence of BKV infection in LVEC. **A.** Live cell images of mock and BKV inoculated LVEC2 at 65dpi. **B.** IF images of LT and VP1 expression in mock and BKV inoculated LVEC2 at 66dpi. **C.** Titration of culture supernatants from mock and BKV inoculated LVEC2 at 62dpi using RPTE1.

The limited infection and lack of cell death in infected VEC prompted us to monitor infected VEC over a longer period. Inoculated LVEC2 were passaged 7 times over a 3-month period. The control mock cells in this experiment were passaged 8 times in 2 months (Fig 6A). While most mock cells appeared to be enlarged and flattened, a morphology indicative of senescence, the BKV inoculated cells showed less morphological changes. Expression of LT and VP1 was observed in infected LVEC2 at 66dpi (Fig 6B). The presence of infectious particles was confirmed by inoculating RPTE1 with the culture supernatants collected from mock and BKV inoculated LVEC2 at 62dpi (Fig 6C). Independently, BVEC were inoculated and maintained for 8 weeks without passaging, at which point the cells continued to show expression of LT and VP1 (S2 Fig). The culture supernatant of BKV infected BVEC at 8-week post infection (wpi) also contained infectious BKV particles as verified by inoculation of RPTE1. These results demonstrate that BKV infection persists in VEC for at least two months.

### BKV infection of epithelial and endothelial cells upregulates genes associated with cell proliferation

To examine the influence of infection on global patterns of gene expression, we performed a series of RNA-seq experiments on BKV-inoculated RPTE and VEC. We first tried several methods of RNA isolation using RPTE, in each case comparing mock to infected cells. These included examining gene expression patterns using total RNA, cytoplasmic or nuclear RNA, as well as polysome-associated RNA. Within each experimental condition, we did not observe significant differences in RNA expression using these different methods (S3 Fig). Therefore, we compared gene expression patterns of mock and infected RPTE and VEC using total RNA.

**Fig 7:**
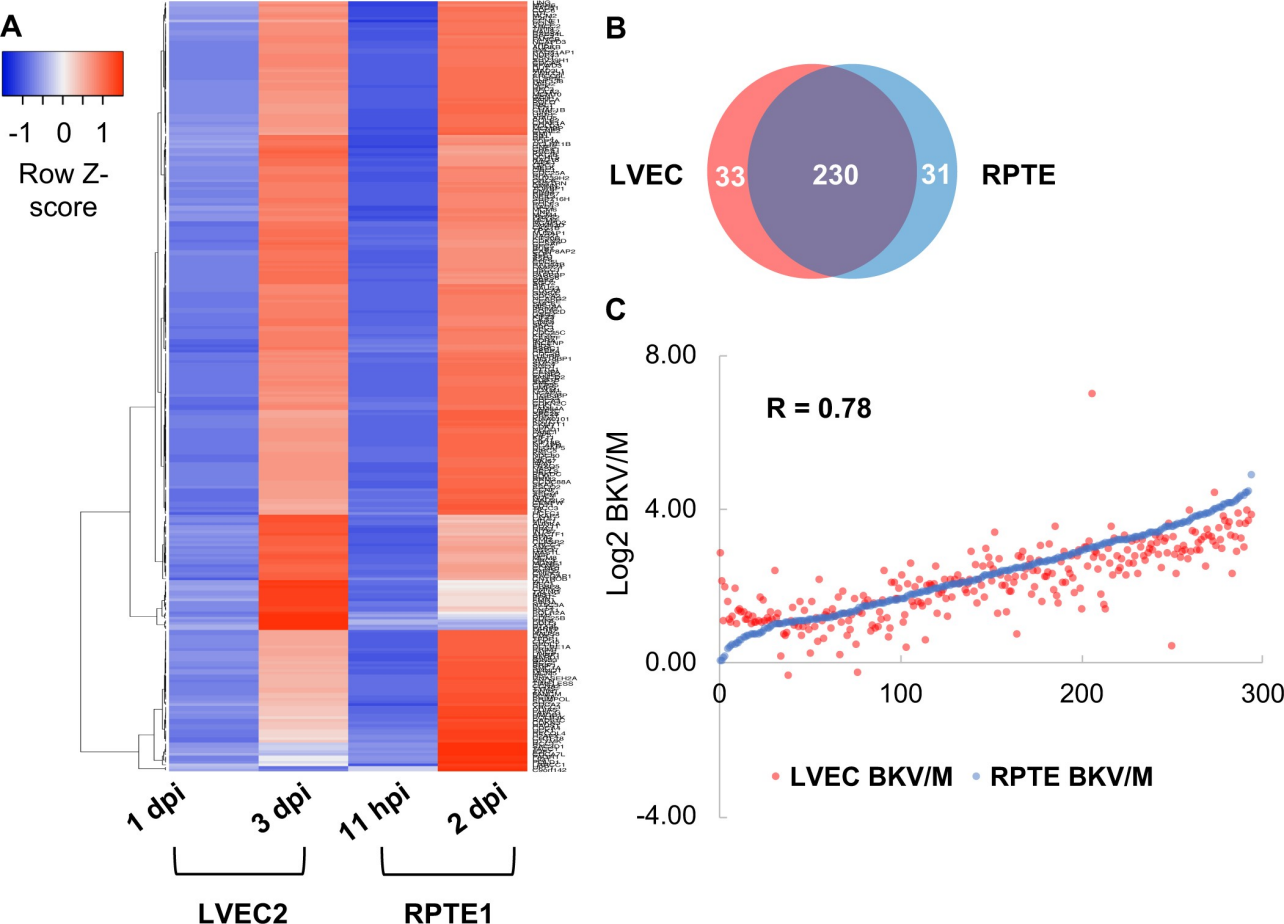
Upregulation of cell proliferation genes in BKV infected RPTE and LVEC. **A.** A heatmap was generated to show upregulation levels (log_2_ BK/M) of cell proliferation genes for RPTE1 and LVEC2 at early and late time points. **B.** The Venn diagram shows the overlap of cell proliferation genes in BKV infected RPTE1 and LVEC2. **C.** Increase (log_2_ BK/M) of cell proliferation genes in BKV infected RPTE1 and LVEC are well correlated. Correlation coefficient (R) is listed on the chart.

We detected very few genes upregulated or downregulated at early time points (11 hpi for RPTE1 and 1dpi for LVEC2) before significant accumulation of viral transcripts occurs (S4 Fig). At later time points, we identified 670 upregulated genes in RPTE1 while 840 were upregulated in LVEC2. The complete lists of upregulated genes with corresponding log ratios can be found in S2 and S3 Tables. The lists of upregulated genes were analyzed using DAVID (https://david.ncifcrf.gov/) for functional classification. This analysis uncovered several functional clusters highly enriched for genes involved in cell proliferation, including cell cycle regulation, cell division, DNA replication and DNA repair. For simplicity we refer to them as proliferation genes. The heat map shown in Fig 7 summarizes the changes in the levels of cell proliferation genes for RPTE1 and LVEC2. Upregulation of most genes appeared to be very similar in both cell types at the two later time points (Fig 7A). Indeed, out of the approximately 260 upregulated proliferation genes in RPTE1 and LVEC2, 230 were common between the two cell types (Fig 7B). In addition, the level of upregulation of these genes showed a strong positive correlation (Fig 7C). These results demonstrate a robust increase of cell proliferation genes by BKV infection in both RPTE and VEC. We also identified 53 and 97 downregulated genes in RPTE1 and LVEC2, respectively, at the later time points. Analysis using DAVID showed no significant enrichment for functional clusters.

### BKV infection of vascular endothelial cells results in increased mRNA levels of many interferon-stimulated genes

**Fig 8:**
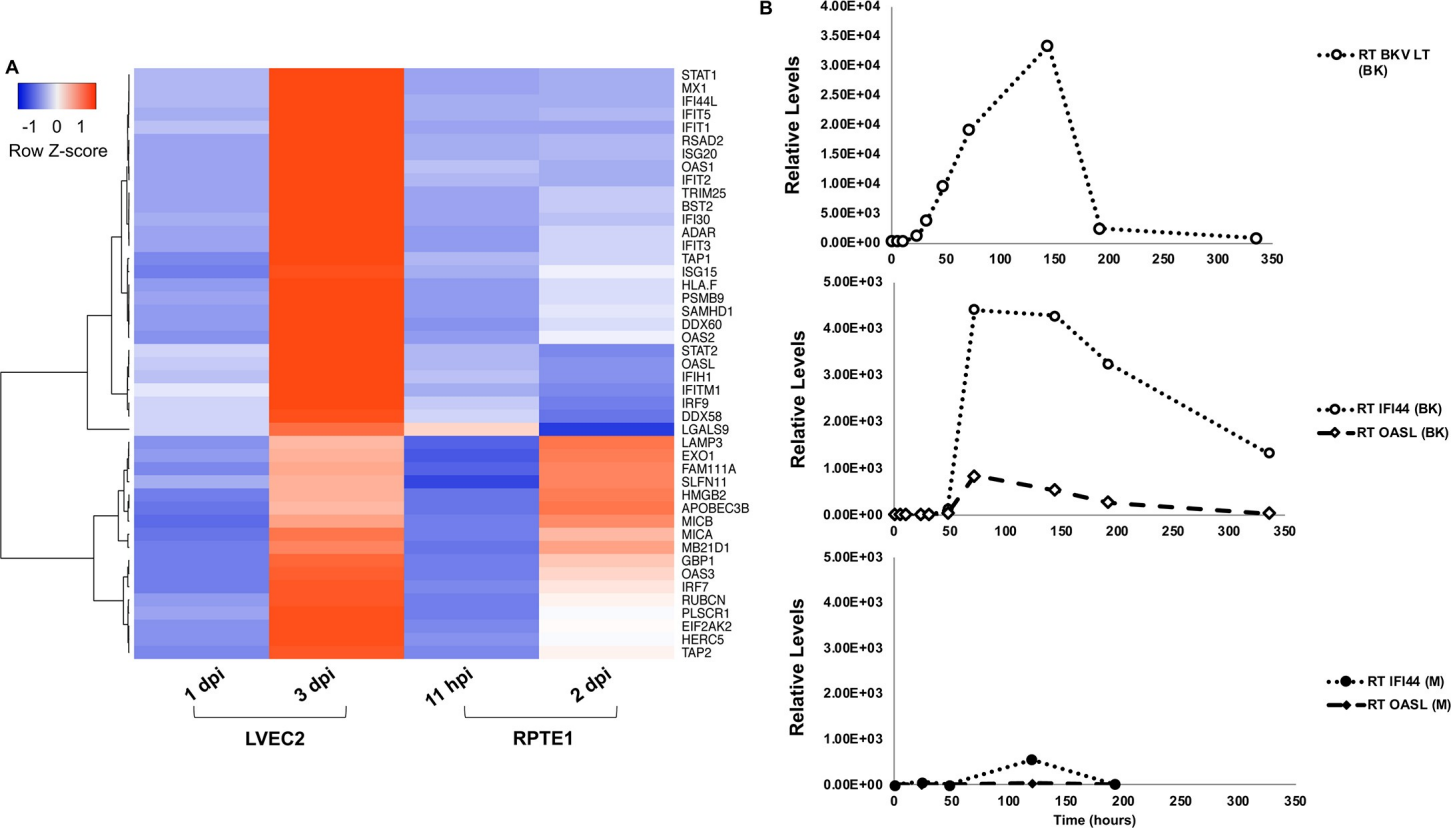
BKV infection induced ISGs in VEC, but not RPTE. **A.** Heatmap representation of ISGs and additional genes involved in immunity using levels of upregulation in log_2_ BKV/M ratios. **B.** Expression of IFI44 and OASL in BKV infected BVEC took place after LT expression level rose. RT-qPCR analysis was used to measure mRNA levels of LT, IFI44 and OASL. Top panel, LT in BK-inoculated cells; Middle panel, IFI44 and OASL in BK-inoculated cells; Bottom panel, IFI44 and OASL in mock cells.

In addition to the cell proliferation genes commonly upregulated in both cell types, functional classification analysis revealed a group of genes involved in type I IFN signaling induced specifically in LVEC2 (Fig 8A). Genes in this cluster include many well characterized ISGs (interferon stimulated genes), such as antiviral effectors [MX1, RSAD2 (Viperin), BST2 (tetherin), ISG15, HERC5, EIF2AK2 (PKR), OAS1, 2, 3 and OASL] and pathogen sensors [DDX60, DDX58 and MB21D1 (cGAS)], as well as positive regulators of IFN signaling [STAT1 and 2, and IRF 7 and 9]. Additional genes involved in antigen presentation, including HLA-F, MICA and MICB, PSMB9 (LMP2), TAP1 and 2, were also upregulated in BKV inoculated LVEC2. Elevated levels of MICB and cGAS were also seen in BKV inoculated RPTE1 but the majority of ISGs were not induced in this cell type (Fig 8A).

Using RT-qPCR, we monitored the induction of two ISGs in BVEC, IFI44 and OASL, during a 2-week time course. Levels of both transcripts increased dramatically in BKV infected BVEC between 2 and 3dpi (Fig 8B, middle panel). Expression levels of both ISGs appeared to peak at 3dpi, remained elevated until 6dpi and then gradually decreased. Similar trends were observed for ISG56 and OAS1 although the level of induction was less robust. The level of IFI44 was maintained at higher than basal level until about 8dpi. The kinetics of IFI44 and OASL upregulation observed in this experiment are similar to the pattern of LT expression (Fig 8B, top panel), which rose dramatically between 1 and 6dpi and then decreased. A slight upregulation of IFI44 was observed in mock at 5dpi (Fig 8B, bottom panel), but the increase was considerably lower than the levels seen in BKV infected samples. Our results establish that BKV infection in VEC, but not in RPTE, leads to a robust and lasting activation of ISGs.

### BKV infection of vascular endothelial cells triggers interferon β production and activation of IRF3 and STAT1

**Fig 9:**
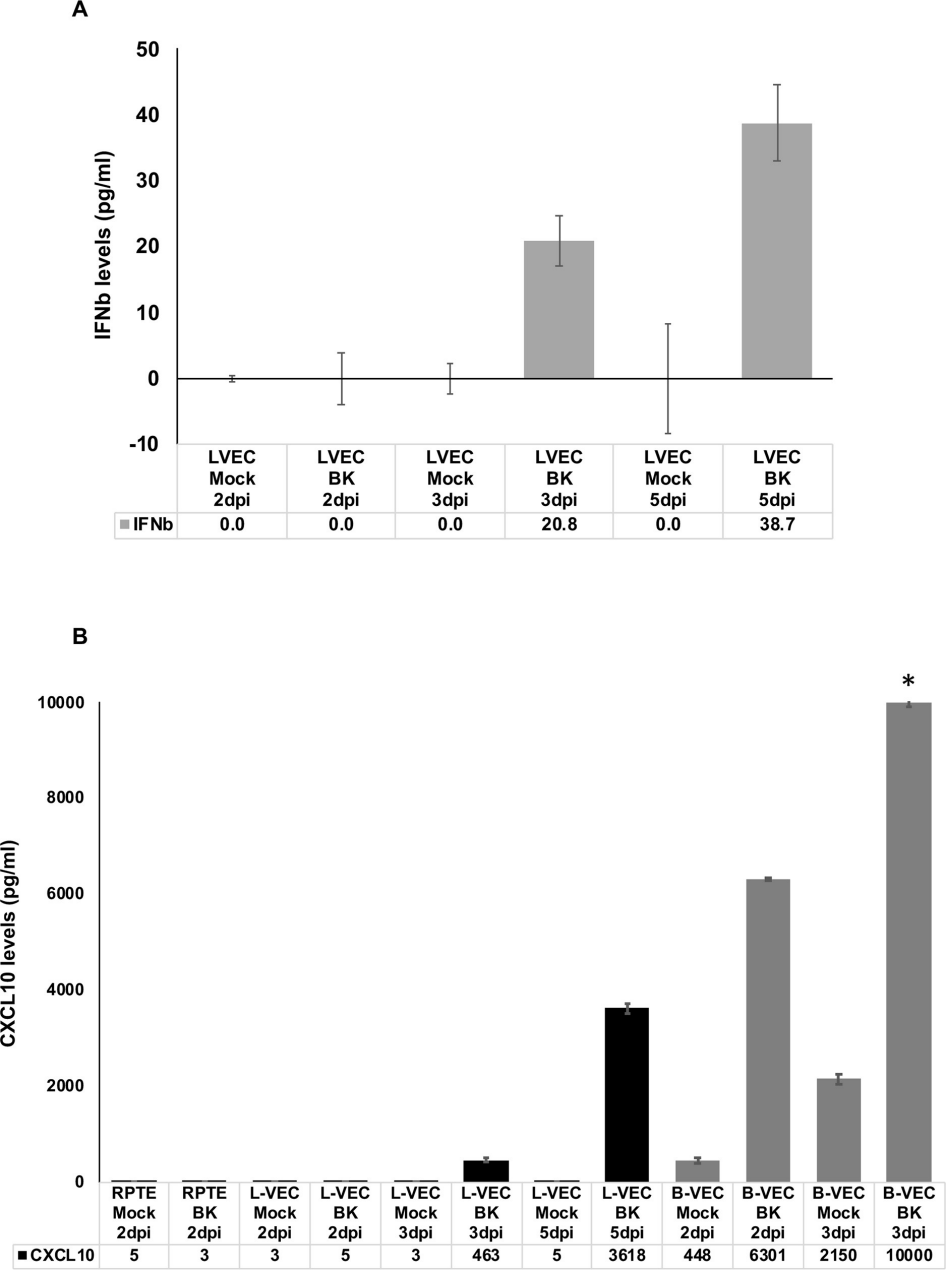
BKV infection of vascular endothelial cells triggers interferon β production and secretion of CXCL10. Supernatants from mock or BKV-infected cultured RPTE, BVEC and LVEC cells were collected at the specified points after infection and evaluated for their levels of different cytokines. Cytokine levels for each cell type and condition tested are represented in the graphs. **A**. Production of IFNβ was increased in BKV-infected LVEC at 3 and 5 dpi. **B**. Production of CXCL10 (IP-10) was increased upon BKV-infection in both BVEC and LVEC endothelial cells, but not in RPTE cells. Error bars represent standard deviation for three measurements.

The observed induction of multiple ISGs led us to hypothesize that BKV infection of VEC results in production of IFN that triggers activation of IRF3 and assembly/nuclear translocation of ISGF3, which subsequently leads to induction of ISGs. We thus tested the levels of various cytokines in culture supernatants from mock and BKV infected RPTE and LVEC at various timepoints. Low levels of IFNβ were found in supernatants from BKV infected LVEC at 3 and 5 dpi (Fig 9A). IFNβ was not detectable at early time points in BKV infected LVEC nor in BKV infected RPTE up to 7dpi. In addition, we identified dramatic increases of the chemokine CXCL10 in BKV infected LVEC and BVEC but not in RPTE using a Luminex assay (Fig 9B). We did not detect production of IFNα or γ.

**Fig 10:**
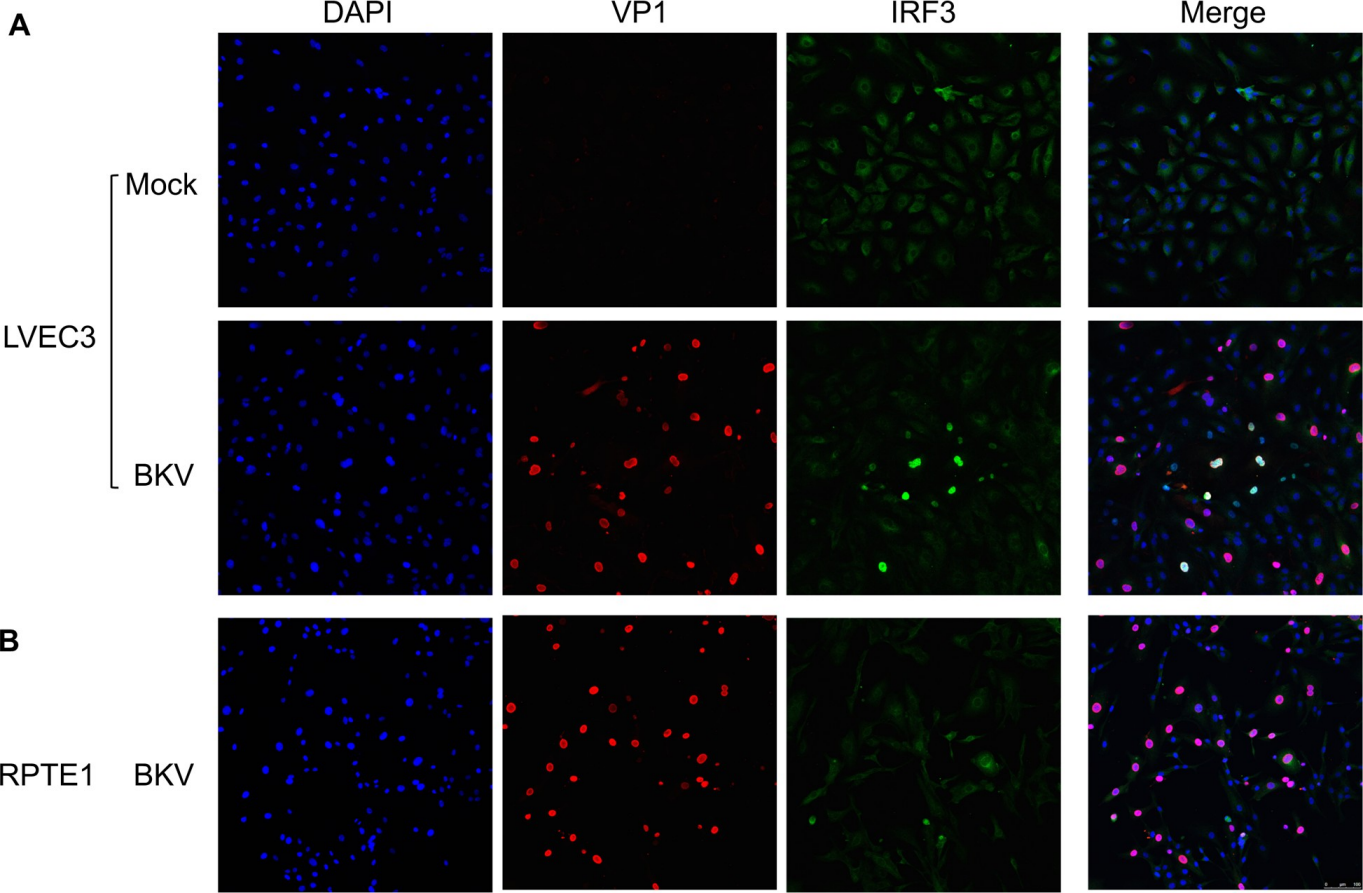
Nuclear translocation of IRF3 in BKV infected LVEC. **A.** Double staining of VP1 and IRF3 showed nuclear localization of IRF3 in BKV inoculated (lower panel) but not mock (upper panel) LVEC3. **B.** Nuclear IRF3 was not detected in BKV infected RPTE1 by IF. Staining of VP1 and IRF3 are shown separately and then as merged images.

**Fig 11:**
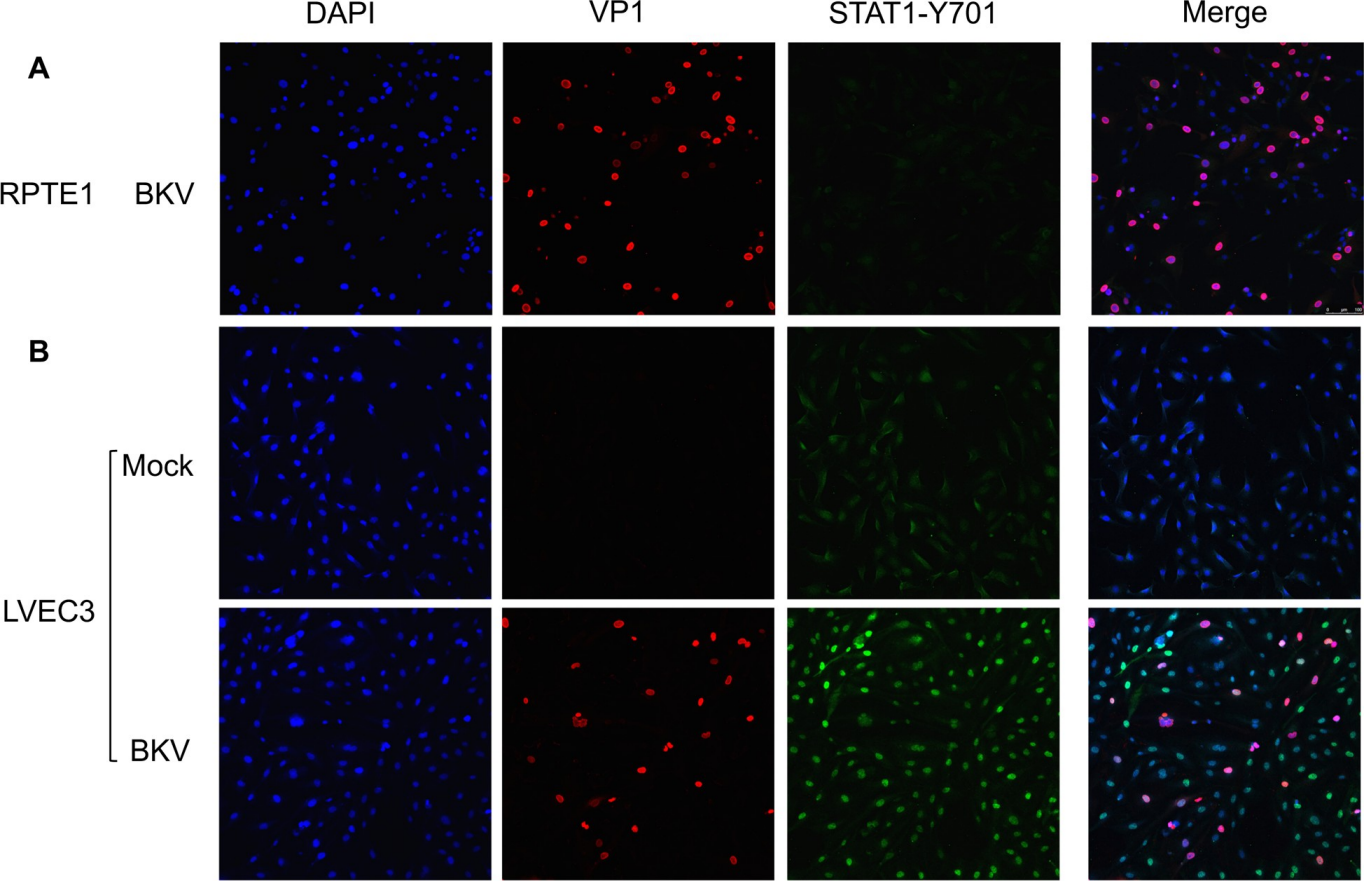
Activation of STAT1 in BKV infected LVEC. **A.** No nuclear STAT1 was detected in BKV inoculated RPTE1 by IF. **B.** Double staining of VP1 and STAT1-Y701 showed nuclear translocation of STAT1 in BKV inoculated (lower panel) but not mock (upper panel) LVEC3. The staining of VP1 and STAT1-Y701 are shown separately and then as merged images.

We then examined two key events in the IFN signaling pathway, nuclear translocation of IRF3 and phosphorylation/nuclear translocation of STAT1 using IF. Co-staining with VP1 and IRF3 antibodies showed the scattered presence of nuclear IRF3 in BKV inoculated but not mock LVEC3 (Fig 10). However, not all VP1 positive cells showed nuclear IRF3 staining. Quantification indicated 54% of nuclear IRF3 positive cells co-stained with VP1 and 27% of VP1 positive cells co-stained with nuclear IRF3. While low background staining of IRF3 was observed in mock, the signal was excluded from the nucleus (Fig 10A, top panel). Similarly, no nuclear staining of IRF3 was observed in BKV inoculated RPTE1 (Fig 10B). We then examined the presence of activated STAT1 with STAT1-Y701 (STAT1-phospho-tyrosine 701) antibody. While RPTE cells were able to respond to IFNβ treatment by translocating STAT1 to the nucleus (S6 Fig), BKV inoculated RPTE1 showed no signs of STAT1-Y701 (Fig 11A). In contrast, we observed nuclear translocation of phosphorylated STAT1 in all BKV inoculated LVEC3, independently of VP1 expression (Fig 11B), suggesting that paracrine signaling may be responsible for the uniform activation of STAT1. These results are consistent with the presence of secreted IFNb in the supernatant of BKV-inoculated LVEC (Fig 9A) and further support activation of an antiviral response in BKV infected VEC.

## Discussion

Most BKV productive infections are observed in the kidneys of immunosuppressed patients. The fact that healthy individuals occasionally secrete BKV particles in urine also suggests that the virus infects cells of the urinary tract. However, the route of initial BKV infection is unknown and little is known about how cells from other organs respond to BKV exposure. To assess the response of different human cells to this virus, we examined the responses of nine different primary human cell types to BKV inoculation. These included epithelial cells, endothelial cells and fibroblasts from the lung, kidney, bladder and ureter. We found that BKV is capable of infecting all of these cell types as assessed by TAg and VP1 expression. Furthermore, we confirmed that progeny virions are produced in RPTE and VEC cultures. Thus, BKV has the ability to infect a wide range of cell types in both the urinary and respiratory systems. This broad tissue/cell type tropism may facilitate dissemination of the virus within the host body and allow BKV to reach specific sites, such as the kidney, to establish a lifelong persistent infection.

All epithelial and fibroblasts cells we tested underwent cell death within two weeks post-infection (Fig 1). In contrast, VECs from the lung and bladder survived for at least eight weeks after the initial BKV inoculation (Fig 6 and S2 Fig). Since uninfected RPTEs can be maintained for up to 2 months in culture without apparent morphological changes, persistence of BKV infection in VECs is not likely due to intrinsic higher stability and/or greater longevity of primary endothelial cells in culture. In addition, infection of VECs was not severely delayed, since we could detect viral DNA replication and progeny virus production as early as 3 days post-infection. Furthermore, we found that the expression of viral proteins and a low-level production of infectious progeny virions were maintained through several passages of primary VEC cultures. These results are consistent with BKV establishing a persistent infection in endothelial cells.

To better understand the underlying causes of the distinct responses by VEC and RPTE to BKV infection, we compared changes in cellular transcriptomics induced by BKV infection in the two cell types by RNA-seq. We observed common upregulation of cell proliferation genes in infected VEC and RPTE. These results concur with the well-established mechanism of LTs of polyomaviruses, which block the cellular retinoblastoma proteins to drive resting cells into the cell cycle and to support viral DNA replication [25–28]. Our results, both indicating upregulation of cell proliferation genes in BKV infected cells and showing little evidence for an active interferon response in BKV-infected RPTE, are in agreement with two previous gene expression profile studies using BKV infected RPTE [20, 23]. In contrast to RPTE, VEC mounted a robust antiviral response to BKV infection resulting in the upregulation of many ISGs and other immune related genes. Interestingly, our results differ from a previous microarray analysis using total RNA collected from BKV inoculated HUVEC at 40 hpi, in which the authors observed upregulation of just two ISGs [29]. The early timing for RNA collection is likely responsible for the lack of ISG induction in this previous study. Indeed, the robust ISG induction shown by our results was observed at 3dpi (Fig 8A) but not 2dpi. Results of RT-qPCR analysis on IFI44 and OASL induction clearly showed that the RNA levels of both ISGs increased dramatically between 2 and 3dpi (Fig 8B). Moreover, IFNβ was not detected until 3dpi in BKV infected LVEC (Fig 9). Nevertheless, heterogeneity between HUVEC and microvascular endothelial cells has been recognized [30–32] and could contribute to the different outcomes of viral infection. A side by side comparison between the two cell types will accurately determine whether they respond to BKV infection differently.

The production of ISGs in response to viral infection is initiated by the production of type I interferon. We detected IFNβ in the media of BKV-infected VECs by ELISA, while uninfected VECs or BKV-infected RPTE produced little or no interferon (Fig 9). Consistent with these results, we detected phosphorylated STAT1 and nuclear IRF3 in BKV-infected VECs while, in infected RPTE, STAT1 remained unphosphorylated and IRF3 remained cytoplasmic (Figs 10 and 11). These results indicate that interferon pathway activation by BKV is cell-type specific, as endothelial cells from two different organs and from multiple donors exhibited an interferon response, while renal proximal tubule epithelial cells failed to do so.

Several studies have shown that endothelial cells are important for innate immune signaling. For instance, endothelial cells were identified as the master regulators of cytokine storm during infection with influenza virus [33]; and human cytomegalovirus infection led to a robust type I IFN response dependent on the cGAS-STING signaling in primary human endothelial cells [34]. We also detected secretion of CXCL10 by BKV inoculated VECs from both the lung and the bladder. CXCL10 is a pluripotent chemokine derived from endothelial cells. Through binding to its receptor CXCR3, CXCL10 mediates the recruitment and activation of monocytes, T cells and natural killer cells, and therefore plays critical roles in defense against infectious diseases [35]. Multiple reports have associated CXCL10 to nephropathy and suggested it as a marker for diagnosis and prognosis of the disease [36–40]. Our findings that elevated CXCL10 levels are present in BKV inoculated VEC implicates the potential of these cells as important sources of CXCL10 during pathogenesis of BKV nephropathy.

It remains unclear at this point why VECs mount an antiviral response to BKV infection while RPTEs do not. Several possibilities exist regarding the mechanisms involved in their different responses. In order for cells to mount an innate immune response against a viral infection, the entry and/or replication of the virus has to be sensed. Therefore, it is possible that RPTEs lack the machinery for detection of BKV infection and thus no response is triggered. Alternatively, viruses have evolved to counter cellular defense through various ways either by blocking the activities of antiviral effectors or by targeting them for degradation. The different responses of RPTEs and VECs demonstrated in this work may suggest that BKV has the ability to circumvent any potential innate immune responses mounted by RPTEs but not that by VECs. Further investigation testing the above hypotheses will facilitate our understanding of the interactions between BKV infection and host cells at the mechanistic level.

Based on the organization of their NCCR sequences, where the replication origin and the early and late promoters reside, polyomavirus BKV can be classified into two main groups, the archetype and the rearranged. The archetype BKV is found commonly among healthy individuals but very difficult to propagate *in vitro*, whereas the rearranged BKV is predominantly associated with diseases and can be grown efficiently using cell culture systems [1, 41–43]. The mechanisms underlying NCCR rearrangement and how this rearrangement contributes to BKV pathogenesis are not well understood. To date, most BKV studies have focused on the rearranged type. The Dunlop strain used in this study also belongs to the rearranged BKV group. Recent progress in methods for increasing yield of viral stock have made investigation of the archetype virus more feasible [21, 44], and thus provide the opportunity for future experiments aiming at addressing the consequences of archetype BKV infection in endothelial cells, particularly regarding whether that infection also leads to activation of IFN response.

In conclusion, we have demonstrated that BKV infection elicits a robust IFN response in human microvascular endothelial cells, suggesting that these cells may play essential roles in triggering and mediating antiviral defense against BKV. Furthermore, we found that BKV infection is limited, but not abolished, in endothelial cells, raising the possibility that viral persistence is maintained in this cell-type, while productive infections linked to pathology occurs mainly in epithelial cells. Further studies will be required to establish the molecular basis for this cell type specific response to BKV infection and the role it plays in disease.

## Materials and methods

### Cells, cell culture reagents, viral stock and infection

Human primary cells were purchased from commercial sources and cultured following the manufacturers’ instructions. Details about cell type, supplier, donor information and number of donors tested, as well as specific media used are listed in S1 Table. Although the cells came from apparently healthy human donors, they were not tested thoroughly for presence of viruses, except for the few highly pathological agents monitored by the supplier. We thus took the effort to identify the presence of potential adventitious agents in the RPTE1 and LVEC2 used for our RNAseq analysis, and found no evidence indicating the existence of such agents.

The initial viral stock of BKV was provided by Dr. Michael Imperiale. Inoculation with BKV for infection was carried out following protocols previously described [19]. Briefly, BKV viral stock was diluted in growth media and added to cells precooled to 4°C. Cells were incubated for 1 hour at 4°C with gentle rocking every 15 to 20 minutes. The media containing viral stock were removed after the incubation. Cells were washed once with 1X PBS, supplemented with fresh growth media and then returned to the CO2 incubator.

To propagate the virus, RPTE were inoculated at a MOI of 0.1. Most cells died by 12 to 14dpi. The supernatant was collected and frozen at -20°C until processed. For preparation of the viral stock, the supernatant was first cleared by centrifugation at 8000g for 30 minutes. The cleared supernatant was layered on top of a 20% sucrose solution and centrifuged at 25,000rpm for 3 hours at 4°C. The virus pellet was resuspended in Buffer A (10mM HEPES, pH 8, 1mM CaCl_2_, 1mM MgCl_2_ and 5mM KCl), followed by centrifugation at 13,000rpm for 5 minutes at 4°C to remove any particulate debris. This viral stock was then aliquoted and stored at -20°C. We initially prepared viral stock by collecting both the supernatant and cell debris, which was then treated by neuraminidase and detergent following established protocols [19]. These additional steps for extracting viruses from cell debris did not result in any dramatic increase of the virus yield in RPTE cells. We therefore switched to preparing viral stock using only the infection supernatant.

Titration of virus was performed by inoculating RPTE1 cells with serial dilutions of either viral stocks or infection supernatants as described above. Cells were fixed at 2dpi and stained with an antibody against LT. Quantification of % cells infected was performed via ImageJ by determination of % LT positive cell within the entire population of cells visualized by DAPI staining. A titer was calculated using the equation below:

$$Titer\;(FFU/ml)\;=\;\frac{Number\;of\;cells\;*\%\;Cells\;infected\;*Dilution\;factor}{Volume}$$

To monitor cell death in RPTE and LVEC, the inoculation was set up in 12-well plates. For each data point, the average cell number and standard deviation were calculated using cell counts from 3 separate wells.

Recombinant human IFNβ (R&D Systems) was used to treat RPTE at the concentration of 100IU/ml for 3 hours before fixation.

### Immunofluorescence and microscopy

The following primary antibodies were used in this study, Pab416 (LT antibody, mouse monoclonal Ab, 1:100) [45], GaT (Goat anti-full-length LT protein polyclonal Ab that reacts with both LT and sT, 1:400), anti-VP1 (Abnova, mouse monoclonal Ab, 1:400), rabbit anti STAT Y701 (Cell Signaling, 1:400) and rabbit anti IRF3 (Cell Signaling, 1:400). We used the following secondary antibodies, goat anti mouse FITC conjugated (Sigma, 1:200), goat anti mouse Alexa Fluor 488 or 568 (Thermo Fisher Scientific/Invitrogen, 1:400), or goat anti rabbit Alexa Fluor 488 (Thermo Fisher Scientific/Invitrogen, 1:400), chicken anti goat FITC (Thermo Fisher Scientific). Incubation of primary antibodies was performed at 4°C for overnight. Incubation of cells fixed in coverslips with all secondary antibodies was performed for 1 hour at room temperature. All epithelial and endothelial cells were examined by staining with Pab416 and anti-VP1 separately, except for fibroblasts from lung and bladder that were co-stained with GaT and anti-VP1.

Except for the incubation with primary antibodies, all other steps in immunofluorescence were carried out at room temperature. Cells on coverslips were fixed in 4% formaldehyde (Sigma) for 20 minutes and washed three times with 1x PBS. For single staining with Pab416 or anti-VP1, cells were permeabilized with 0.1% TritonX-100 for 5 minutes and washed three times with 1x PBS. Coverslips were then blocked with 5% goat serum in 1XPBS for 1 hour. Secondary antibody for these coverslips were goat anti mouse FITC. For co-staining of GaT and anti-VP1, coverslips were also permeabilized in 0.1% TritonX-100, then blocked with 5% normal chicken serum and 5% new born calf serum. The secondary antibodies for GaT and anti-VP1 co-staining were chicken anti goat FITC and chicken anti mouse TRITC. Co-staining using IRF3 or STAT1-Y701 were performed following instructions from Cell Signaling Technology. The coverslips were washed 3 to 5 times using 1X PBS after primary and secondary antibody incubation. After the final wash, the coverslips were dried and mounted with ProLong Gold Antifade reagent with DAPI (Thermo Fisher Scientific/Invitrogen) and imaged using either a Zeiss AX10 microscope with LED camera and a set of fluorescence filters, or a Leica TCS SP5 confocal/multi-photon imaging system.

To quantify % LT positive RPTE and VEC at 2 and 5dpi, the inoculation was set up using cells on coverslips in 12-well plates. For each data point, the average cell number was calculated using cell counts from 3 separate coverslips.

### Isolation of Viral DNA

The QIAprep Spin Miniprep kit (QIAGEN) was used to isolate BKV genomic DNA. The mock and BKV inoculated cells (from 1 well of a 6-well plate/condition) were harvested in 250μl of buffer P1 and then processed following manufacturer’s instructions. The circular BKV genomic DNA harbors a single BamHI recognition site and was linearized by BamHI restriction digestion. BamHI digested pBKV, a plasmid containing the full-length BKV genomic DNA (Genbank accession number, KP412983), was used as positive control. The sequence of pBKV was verified by Sanger sequencing and MiSeq. The digested DNA was resolved on 0.8% agarose gel.

### RNA isolation, RT-qPCR and RNA-seq

Total RNA from whole cells growing in monolayers was first extracted with TRIzol (Ambion), and then purified with Direct-Zol columns (Zymo Research) according to the manufacturer’s instructions. Cytoplasmic and nuclear RNA was isolated using the PARIS kit (Thermo Fisher Scientific) following manufacturer’s instructions.

Isolation of polysome associated RNA was based on previously published methods [46] and suggestions from J. Woolford’s laboratory (Carnegie Mellon University, Pittsburgh PA). Control or BKV inoculated cells (MOI = 4) were allowed to grow in 10 cm diameter dishes to 2dpi. Ten plates of each condition were used for each extraction. After removing the culture media, ribosomes were frozen on mRNA by adding to each plate 1ml of ice-cold lysis buffer (Tris HCl 100mM pH 7.5; NaCl 100mM; Mg2Cl 10mM; DTT 5mM; NP40 0.5%) containing 100 μg/ml of cycloheximide, and incubating the cells on ice for 5 min. Cells were collected with a scraper and the extracts were kept on ice for the entire duration of the procedure. After adding RNase-free DNase (2 μg/ml) and incubating the mixture at 4°C for 20 minutes, the extracts were centrifugated at 14K, 4°, 5 min to remove debris. Total RNA from a portion of each supernatant (0.5 ml) was obtained with TRIzol and Direct-Zol columns (Zymo Research), following the manufacturer’s instructions. The rest of each supernatant was poured over 12.5 ml of a sucrose cushion (60% sucrose in lysis buffer) in Ti70 ultracentrifuge tubes, the tubes were filled with lysis buffer, and samples were centrifuged for 25 hours at 27,000 rpm, 4°C in a Ti70 rotor (Beckman Coulter). The pellets from centrifugation, containing both ribosomes and polysomes, were resuspended in (~0.2ml) of lysis buffer and RNA was immediately extracted with TRIzol and Direct-Zol columns as mentioned above.

RT-qPCR analysis was performed at the Genomics Research Core of the University of Pittsburgh with the *Power* SYBR Green RNA-to-CT 1-Step Kit (Thermo Fisher). All samples were analyzed in triplicate and the values were normalized against those of the corresponding endogenous control (GAPDH). Relative levels indicated in the graphs refer to each specific value relative to the signal observed in mock at 6 hours.

The following sets of primers were used to detect the presence of: BKV LT (5’- GAGTAGCTCAGAGGTGCCAACC and 5’-CATCACTGGCAAACATATCTTCATGGC); BKV sT (5’- GATCTAAAGCTTTAAGGTGCCAACCTATGG and 5’-CATCACTGGCAAACATATCTTCATGGC); GAPDH (5’-AAGGTGAAGGTCGGAGTCAA and 5’-AATGAAGGGGTCATTGATGG); IFI44 (5’-TGCAGAGAGGATGAGAATATC and 5’- ACTAAAGTGGATGATTGCAG); ISG56 (5’-CAACCAAGCAAATGTGAGGA and 5’- AGGGGAAGCAAAGAAAATGG); OAS1 (5’-ATAAAAGCAAACAGGTCTGG and 5’-TCTGGCAAGAGATAGTCTTC) and OASL (5’-AGGGTACAGATGGGACATCG and 5’—AAGGGTTCACGATGAGGTTG).

Paired-end (2x 75bp) RNA-seq strand specific libraries were constructed using enriched polyadenylated RNA by the Genomics Research Core facility at the University of Pittsburgh.

### RNA-seq Analyses

The raw reads were analyzed by FastQC (Andrews S. (2010). FastQC: a quality control tool for high throughput sequence data. Available online at: http://www.bioinformatics.babraham.ac.uk/projects/fastqc). No quality filtering was necessary based on the FastQC analysis. Gene expression values in TPM were determined with CLC Genomics Workbench 11 using the Ensembl GRCh38 human genome [47]. To determine cellular gene upregulation by BKV infection in the RNAseq data we used the following definition: the expression level of a gene had to be greater than or equal to 10 TPM in a BKV infected sample, and the fold change of BKV infected over mock had to be greater than or equal to 2 (Log_2_ BKV _(TPM)_ / Mock _(TPM)_ ≥ 1). For downregulation, the expression level of a gene had to be greater than or equal to 10 TPM in a mock infected sample, and the fold change of BKV infected over mock had to be less than or equal to 0.5 (Log_2_ BK_(TPM)_ / Mock_(TPM)_ ≤ -1).

Correlation analysis of gene expression and upregulation was done using the CORREL function in Microsoft Excel. Functional classification was performed using DAVID Bioinformatics Resources 6.8 [48, 49] (https://david.ncifcrf.gov/home.jsp). Heatmaps were generated using the Heatmapper server [50] (http://www.heatmapper.ca/).

### Luminex and ELISA

To test IFN and additional cytokine production, culture supernatants from mock and BKV infected RPTE and VEC at various time points were collected and stored at -80°C until processing. IFNβ levels were determined with the Human IFN-beta DuoSet ELISA kit (R&D Systems) following manufacturer’s instructions. A 38-plex human cytokine Luminex panel (HCYTMAG-60K-PX38, Millipore Sigma) was used to assay secretion of 38 additional cytokines. The collected supernatants were submitted to the Hillman Cancer Center (UHCC) Luminex Core Laboratory affiliated with the University of Pittsburgh Medical Center for the procedures.

## Supporting information

S1 Fig BKV DNA replication in LVEC and RPTE. Agarose gel image of BKV genomic DNA isolated from mock and BKV inoculated RPTE2 (A, upper panel) and LVEC1 (B, lower panel). M, mock; BKV, BKV inoculated; MwM, molecular weight markers; (+), positive control using digested pBKV plasmid, which produced two bands. Upper band (BKV), linearized BKV genomic DNA; Lower band (VBB), plasmid vector backbone.
(TIF)

S2 Fig Infected BVEC express LT and VP1 at 8 wpi. Images for positive staining of LT (Pab416) and VP1 in BKV inoculated BVEC at 8wpi are shown as indicated. The matching DAPI staining for each field are included.
(TIF)

S3 Fig Biotype distribution of RNAs and correlation of gene expression in initial RNA-seq experiments. Five experiments and a total of 10 RNA samples (mock and BKV for each experiment) were evaluated. Cyt, cytoplasmic; Nuc, nuclear. **A.** Distribution of RNA subtypes in percentage. Note that the minimum value of the Y axis is 70%. **B.** Correlation analyses of gene expression levels in mock (left panel) and BKV inoculated cells (right panel). Expression values were corelated to WholeCell BK2 and genes were sorted on X-axis based on expression in WholeCellBK2. The R values (correlation coefficient) are listed below the charts.
(TIF)

S4 Fig Viral gene expression in BKV infected RPTE and LVEC. **A**. Genome map of reference BKV polyomavirus genome with Genbank accession number, NC_001538.1. **B.** IGV graphs showing coverage of BKV genome by reads from RPTE1 and LVEC2 RNA-seq. **C.** Summary table of BKV gene expression (in RPKM) in infected RPTE1 and LVEC2 at early and late timepoints.
(TIF)

S5 Fig Expression of cell specific markers in RPTE and LVEC determined by RNA-seq. Log_2_ TPM values of 6 RPTE markers (**A**) and 6 endothelial cell markers (**B**) were calculated and plotted for mock and BKV inoculated RPTE1 at 2dpi, and mock and BKV inoculated LVEC2 at 3dpi.
(TIF)

S6 Fig Activation of STAT1 in RPTE1 by IFNβ treatment. IF staining using STAT1-Y701 antibody showed STAT1 nuclear translocation in IFNβ treated RPTE1 (lower panel). No STAT1-Y701 staining was detected in the no IFNβ control (upper panel).
(TIF)

S1 Table Donor information and growth conditions for primary human cells. (XLSX)

S2 Table Complete list of upregulated genes in RPTE1 RNAseq with corresponding log ratios. (XLSX)

S3 Table Complete list of upregulated genes in LVEC2 RNAseq with corresponding log ratios. (XLSX)
