# Supplementary figures and images for: Human polyomavirus BKV infection of endothelial cells results in interferon pathway induction and persistence
Source: PLoS Pathog. 2019 Jan 8;15(1):e1007505. doi: 10.1371/journal.ppat.1007505 (PMC6338385; doi:10.1371/journal.ppat.1007505)

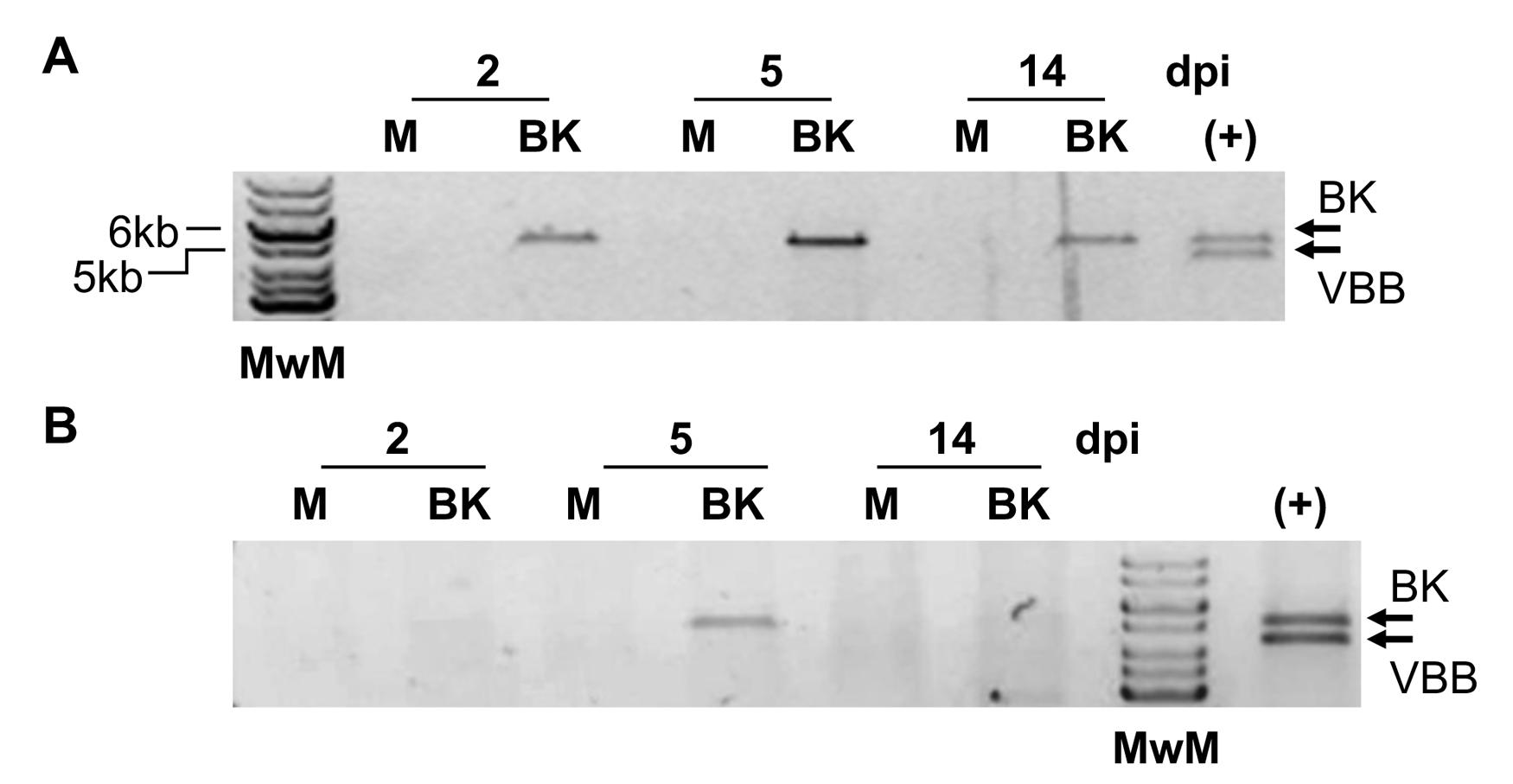

Supplement: S1 Fig — Agarose gel image of BKV genomic DNA isolated from mock and BKV inoculated RPTE2 (A, upper panel) and LVEC1 (B, lower panel). M, mock; BKV, BKV inoculated; MwM, molecular weight markers; (+), positive control using digested pBKV plasmid, which produced two bands. Upper band (BKV), linearized BKV genomic DNA; Lower band (VBB), plasmid vector backbone. (TIF) [file ppat.1007505.s001.tif]

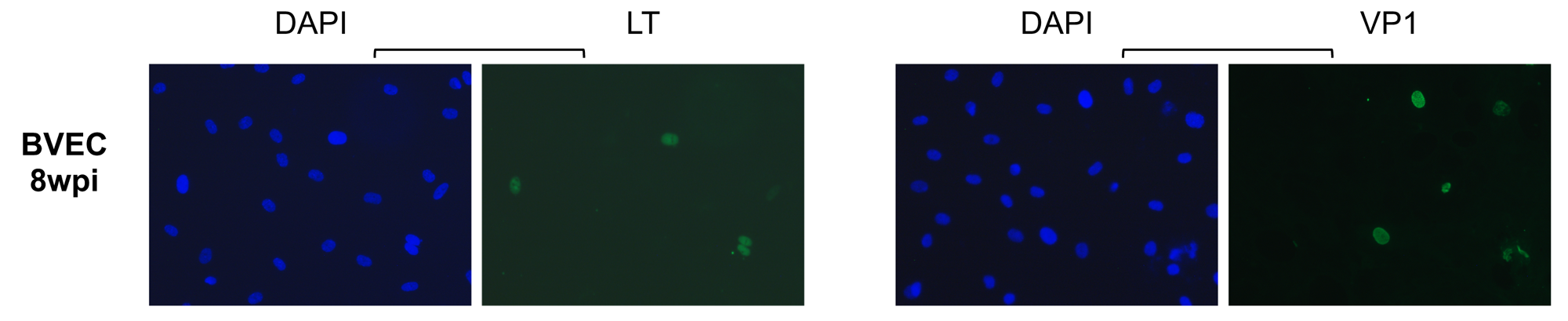

Supplement: S2 Fig — Images for positive staining of LT (Pab416) and VP1 in BKV inoculated BVEC at 8wpi are shown as indicated. The matching DAPI staining for each field are included. (TIF) [file ppat.1007505.s002.tif]

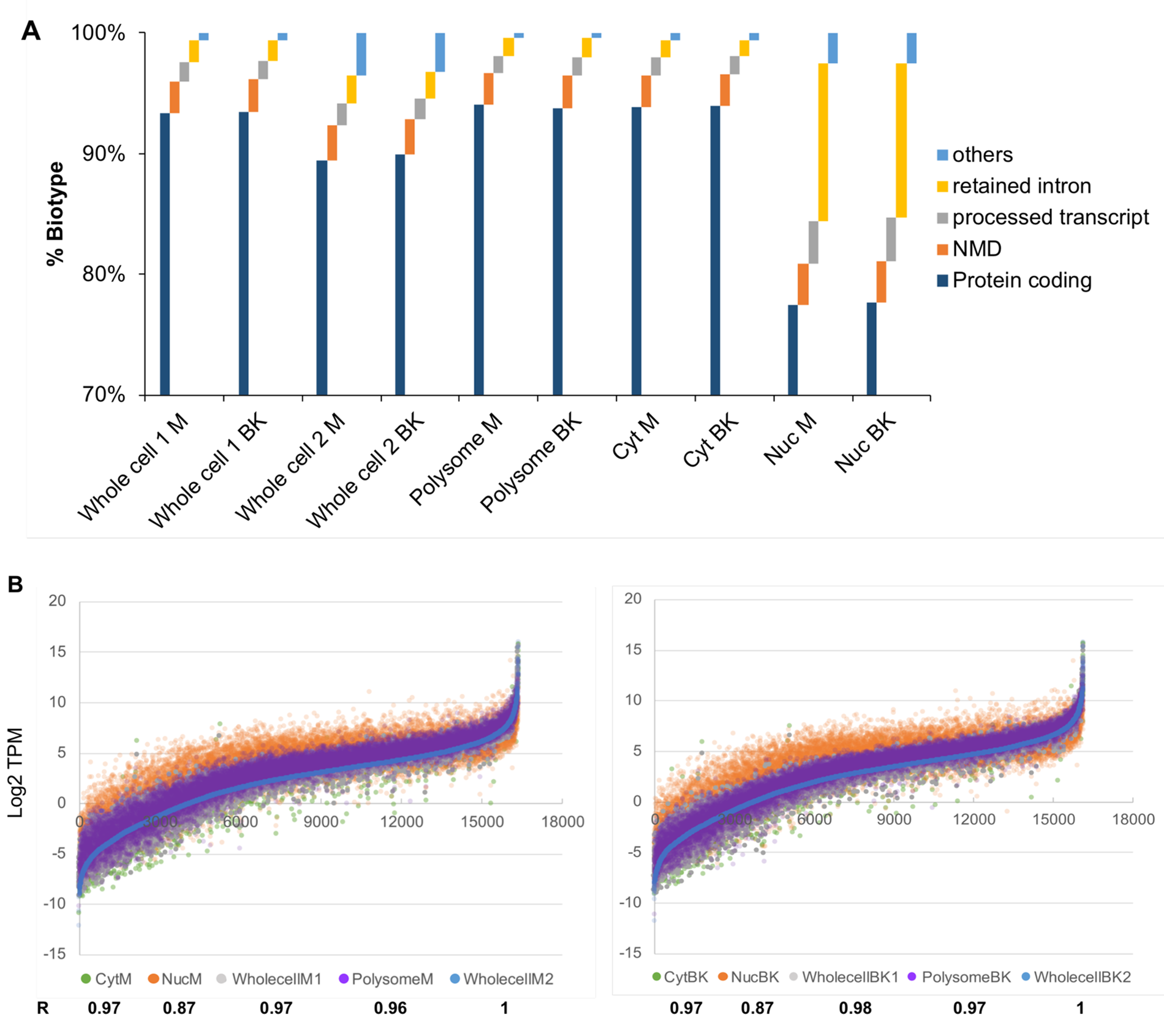

Supplement: S3 Fig — Five experiments and a total of 10 RNA samples (mock and BKV for each experiment) were evaluated. Cyt, cytoplasmic; Nuc, nuclear. A. Distribution of RNA subtypes in percentage. Note that the minimum value of the Y axis is 70%. B. Correlation analyses of gene expression levels in mock (left panel) and BKV inoculated cells (right panel). Expression values were corelated to WholeCell BK2 and genes were sorted on X-axis based on expression in WholeCellBK2. The R values (correlation coefficient) are listed below the charts. (TIF) [file ppat.1007505.s003.tif]

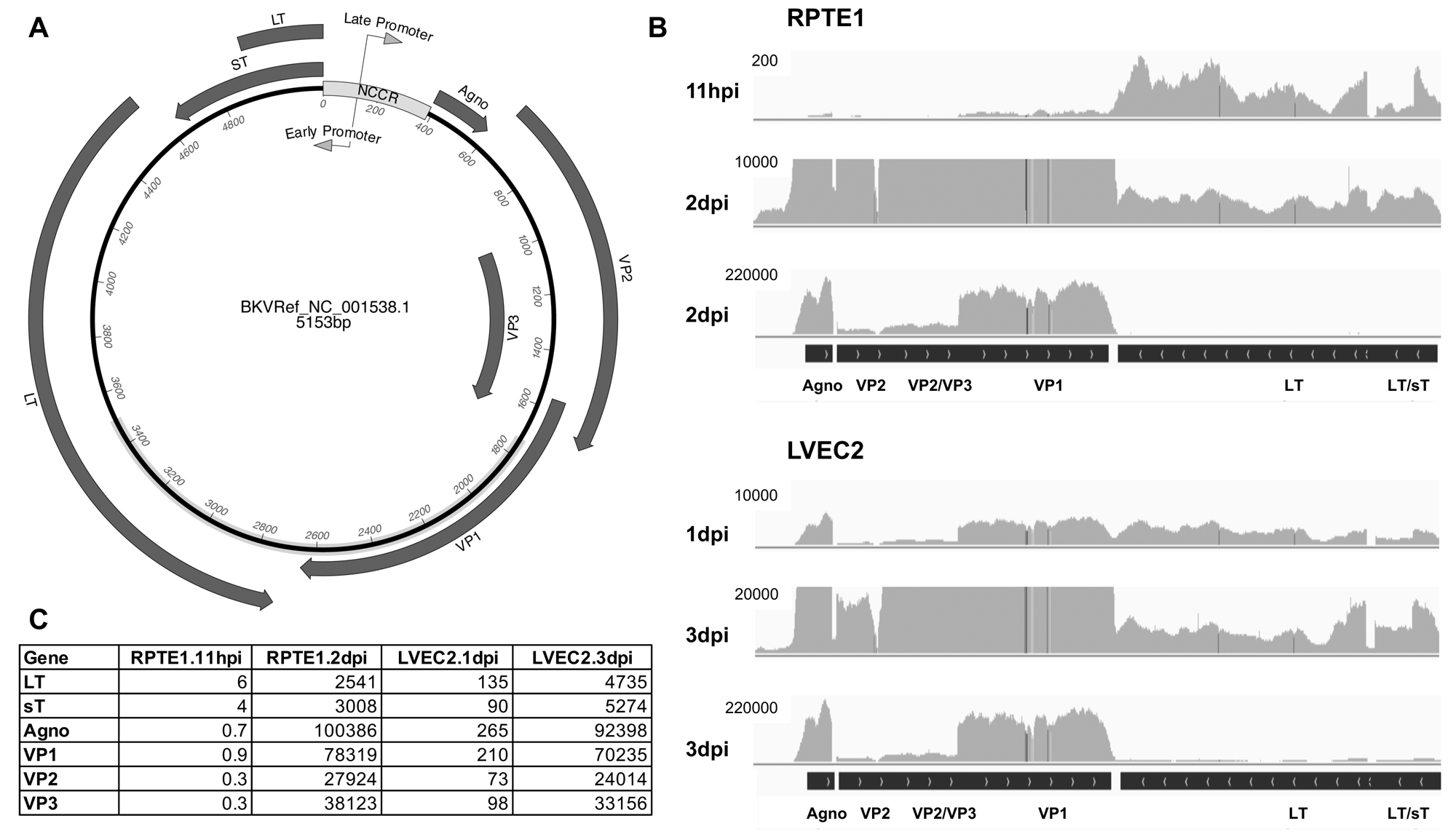

Supplement: S4 Fig — A. Genome map of reference BKV polyomavirus genome with Genbank accession number, NC_001538.1. B. IGV graphs showing coverage of BKV genome by reads from RPTE1 and LVEC2 RNA-seq. C. Summary table of BKV gene expression (in RPKM) in infected RPTE1 and LVEC2 at early and late timepoints. (TIF) [file ppat.1007505.s004.tif]

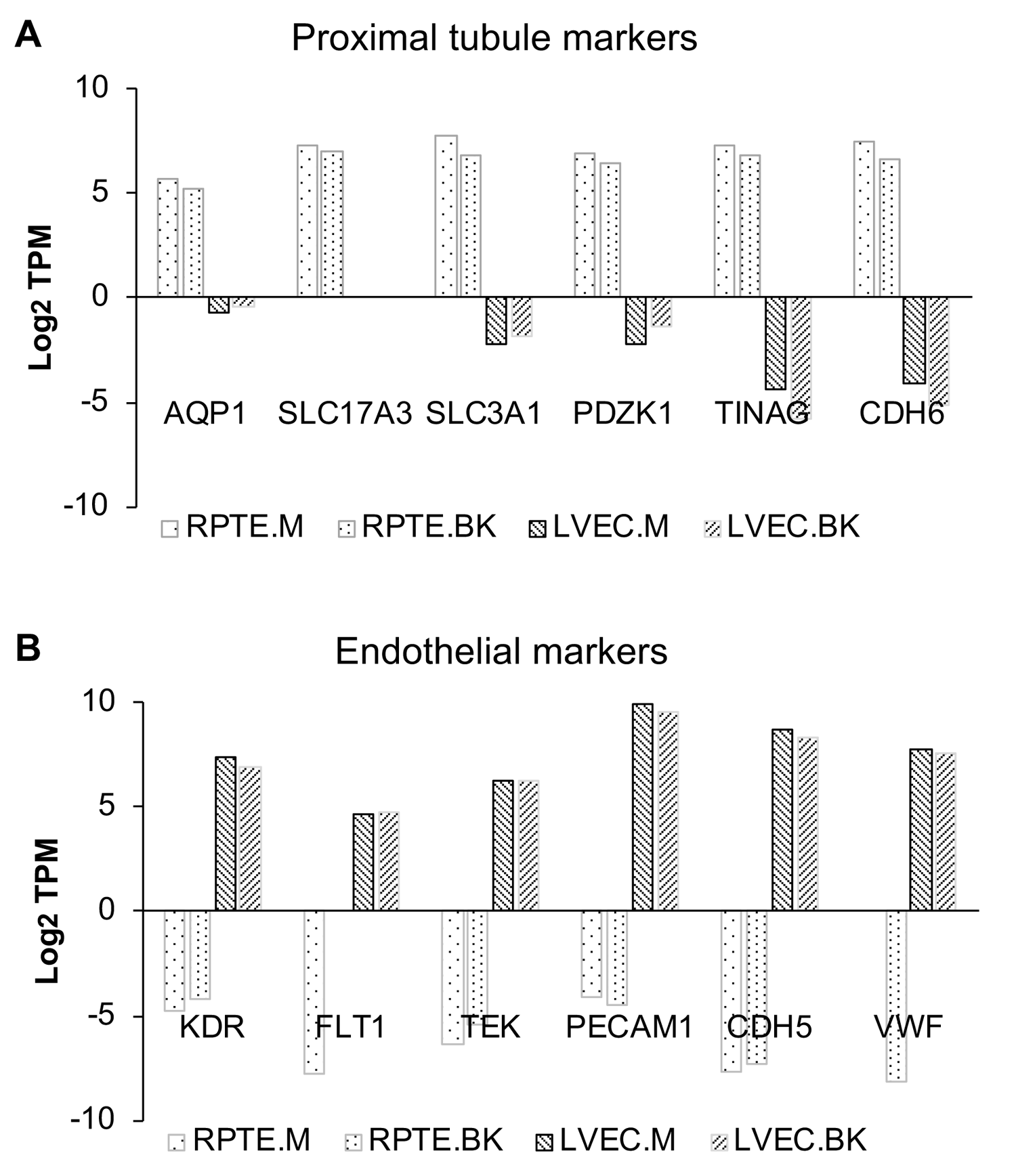

Supplement: S5 Fig — Log2 TPM values of 6 RPTE markers (A) and 6 endothelial cell markers (B) were calculated and plotted for mock and BKV inoculated RPTE1 at 2dpi, and mock and BKV inoculated LVEC2 at 3dpi. (TIF) [file ppat.1007505.s005.tif]

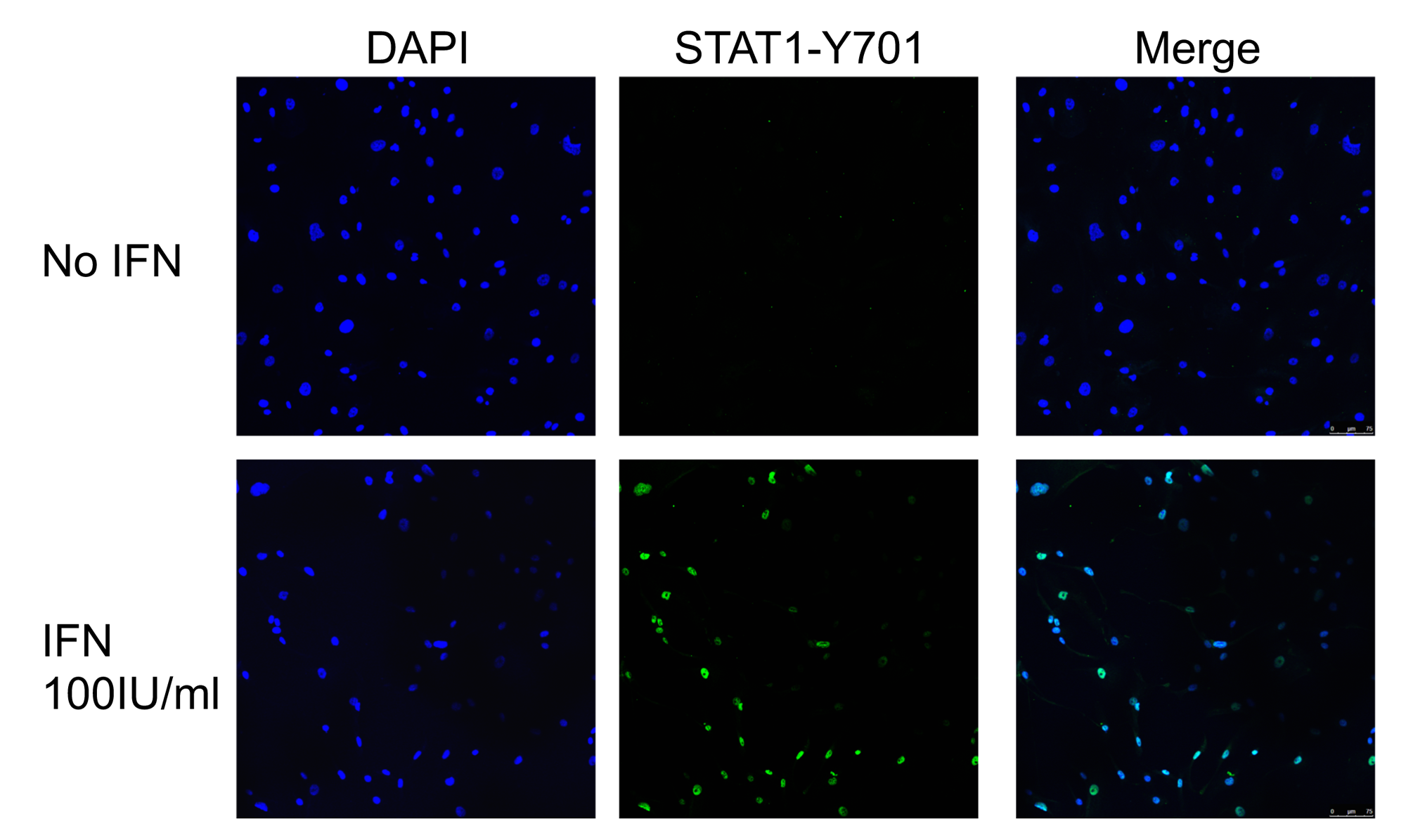

Supplement: S6 Fig — IF staining using STAT1-Y701 antibody showed STAT1 nuclear translocation in IFNβ treated RPTE1 (lower panel). No STAT1-Y701 staining was detected in the no IFNβ control (upper panel). (TIF) [file ppat.1007505.s006.tif]
